# Kinetic modelling of *Chlorella* spp. wastewater treatment and resource recovery in the food industry

**DOI:** 10.1186/s40643-026-01145-x

**Published:** 2026-10-14

**Authors:** Vera Lavelli, Zhi Zhou

**Affiliations:** 1 Department of Food, Environmental and Nutritional Sciences (DeFENS), University of Milan, 20133 Milan, Italy; 2 Lyles School of Civil and Construction Engineering, Purdue University, West Lafayette, IN 47907 USA; 3 School of Sustainability Engineering and Environmental Engineering, Purdue University, West Lafayette, IN 47907 USA

**Keywords:** *Chlorella* spp., Kinetic model, Algal compound, Growth, Phycoremediation, Wastewater valorization, Circular economy

## Abstract

Effluents generated in the food and beverage industry typically contain elevated concentrations of organic carbon sources and inorganic nutrients, representing a significant environmental burden as global food demand continues to rise. Owing to their capacity to effectively assimilate carbon and nutrients while simultaneously generating biomass with potential valorization pathways, *Chlorella* spp. offers a dual benefit of contaminant removal and resource recovery. However, existing algal studies remain predominantly at the laboratory scale, and their competitive advantages have not been demonstrated for full-scale implementation. Kinetic modelling represents an essential tool to elucidate algal growth behavior, optimize operational conditions, and predict performance of algal systems for accurate scale-up, but limited studies are available to comprehensively review kinetic models of *Chlorella* spp. In this review, kinetic models were synthesized, including sigmoidal growth models (Gompertz, logistic, and Verhulst), single-factor and inhibition models (Monod and Andrews), light-dependent photobioreactor models (Huisman), multifactor and interaction models for co-cultures (Lotka–Volterra). Kinetic parameters from these models were compiled to support consistent projections from lab-scale to industrial-scale systems, enabling future techno-economic analysis and life-cycle assessment of algal systems. Other emerging technologies, such as artificial intelligence and machine learning approaches, were also reviewed. The review also identified benchmark processes and outlined a decision tree for developing strategies to enhance resource recovery and phycoremediation performance. Finally, key research needs have been highlighted to guide future work in the field.

**Supplementary Information:**

The online version contains supplementary material available at https://doi.org/10.1186/s40643-026-01145-x.

## Introduction

The food and beverage industry generates effluents characterized by high carbon (C), nitrogen (N), and phosphorus (P) loads. For example, the chemical oxygen demand (COD) in the effluent of tequila processing water can reach up to 132,120 mg·L^−1^ (Najar-Almanzor et al. 2024), total nitrogen (TN) can reach up to 1,900 mg·L^−1^ in poultry slaughterhouse wastewater (Pérez-Guzmán et al. 2024), and total phosphorus (TP) in cheese processing effluents can achieve up to 2,800 mg·L^−1^ (Najar-Almanzor et al. 2024). In the context of increasing global food demand (Nzayiramya et al. 2025), the development of effective treatment solutions for these effluents is urgently needed.

Conventional biological wastewater treatment technologies are robust but generate large amounts of waste sludge without enabling resource recovery (Ahuja et al. 2024). As an alternative, microalgae-based phycoremediation exploits the natural ability of microalgae to assimilate C, N, and P as nutrients (Fernández et al. 2021). Beyond contaminant removal, microalgae cultivation has attracted attention due to its potential to produce high-value bioproducts, including pharmaceuticals (Kaur et al. 2025), food ingredients (Chen et al. 2022; Karabulut et al. 2024), animal feeds (Picknell et al. 2025), biofertilizers, biopesticides, and biofuels (Fernández et al. 2021). Among the various microalgae that are suitable for wastewater treatment, *Chlorella* spp. stands out due to its rapid growth rate, resilience to harsh environmental conditions , and established safety as a source of food ingredients (Safi et al. 2014; Chen et al. 2022; Bilgin et al 2026). *Chlorella*’s resilience to microbial contamination is a crucial aspect that could be managed through eco-friendly approaches such as the use of biofabricated 5-aminolevulinic acid (Lee et al. 2022). In general, *Chlorella*’s traits make it especially promising for applications within food industry facilities. Integration of microalgae into treatment systems directly supports Sustainable Development Goals (SDGs) 6 (Clean Water and Sanitation), 9 (Industry, Innovation and Infrastructure), and 12 (Responsible Consumption and Production).

Several factors may affect the performance of algal systems for wastewater treatment and resource recovery in the food and beverage industry. Light availability governs the transitions among photoautotrophic, heterotrophic, photoheterotrophic, and mixotrophic metabolic pathways (Yeh and Chang 2012). The chemical nature of C, N, and P sources affects biomass yield and biochemical composition (Ma et al. 2022). The choice of cultivation mode (batch, fed-batch, semicontinuous, or continuous) further shapes algal productivity. Due to these complications, process modelling is essential to optimize operating conditions and assess the technical and economic feasibility of full-scale implementation of algae in wastewater treatment and resource recovery (Quiroz et al. 2025).

Previous reviews have explored the use of microalgae to treat industrial effluents as a strategy to advance the circular economy (Gupta et al. 2019; Zabochnicka et al. 2022). What remains missing, however, is a concrete pathway to translate existing research findings into full-scale industrial applications of algae. This review is built on the idea that kinetic modelling represents a pivotal bridge between laboratory experiments and industrial applications. By predicting growth behavior, optimizing operating conditions, and guiding process design, kinetic modelling can accelerate the transition toward industrial systems that are aligned with multiple SDGs. Accordingly, this review aims to: (1) analyze kinetic models to optimize *Chlorella* spp. cultivation in the food and beverage industry effluent; (2) extrapolate laboratory-scale kinetic data to industrial scenarios and assess their potential performance; (3) examine emerging approaches based on artificial intelligence (AI) and machine learning (ML); (4) develop an integrated optimization model.

## Kinetic models of algal growth in monoculture

Cell growth under batch conditions is typically evaluated by plotting biomass, such as cell concentration or optical density, against cultivation time (t). The resulting curves have been described using several models, including the modified Gompertz model (equation (Eq.) 1 and Eq. 2), the logistic model (Eq. 3), and the Verhulst model (Eq. 4) (Zwietering et al. 1990; Padri et al. 2022). The details of these models are shown in Table 1.

Both the Gompertz and logistic models include three biologically meaningful parameters that correspond to the main stages of algal growth: 1) the lag phase, during which cells adapt to the substrate and growth rate is zero; 2) the exponential phase, where the growth rate reaches its maximum; 3) the stationary phase, during which growth progressively declines due to nutrient depletion or accumulation of inhibitory compounds and eventually reaches an asymptotic maximum cell concentration. Accordingly, these models estimate the lag time or the inflection time (λ in eqs. 1–2 or λ’ in Eq. 3, Table 1), the maximum specific growth rate (μ_max_), and the asymptotic biomass concentration (Y_max_) (Zwietering et al. 1990). The Verhulst equation, although simpler, provides only two parameters (μ_max_ and Y_max_) (Padri et al. 2022).

In many studies involving microalgae grown on food processing wastewaters, the growth curve was not modeled. Instead, the specific growth rate (μ in Eq. 5) was calculated from initial and final biomass values as if the culture remained in exponential growth throughout the batch period. However, analyzing the full growth profile is essential to understanding the physiological stages of the culture. Moreover, accurate estimates of the maximum biomass concentration and μ_max_ obtained from sigmoidal models are needed to determine biomass productivity (δY/δθ in Eq. 6, Table 1) as a function of hydraulic retention time (θ in Eq. 6, Table 1) in continuous systems and to predict the optimal retention time (θ_opt_ in Eq. 7, Table 1) (Ruiz et al. 2013).

**Table 1:** Kinetic models of algal cultivation in effluents of the food and beverage industry

Kinetic model	Equation	Reference
Biomass—Modified Gompertz $Y={Y}_{max}\cdot exp\left[-exp\left[\frac{e\cdot {\mu}_{max}\cdot \left(\lambda -t\right)}{{Y}_{max}}+1\right]\right]$ Y (cell⋅mL^−1^), cell concentration at time t; Y_max_ (cell⋅mL^−1^), asymptotic cell concentration; t (d), time λ (d), lag time defined as the x-axis intercept of the tangent drawn at the inflection point; μ_max_ (d^−1^), maximum specific growth rate	(1)	(Blanco et al. 2021; Carbonell Sorí et al. 2025; Kothari et al. 2024; López-Pacheco et al. 2019; Najar-Almanzor et al. 2024; Pérez-Guzmán et al. 2024; Serejo et al. 2021; Širić et al. 2023)
Biomass – Modified double Gompertz $Y= {Y}_{max1}\cdot {e}^{-e\cdot {\mu}_{max1}\cdot \left({\lambda}_{1}-t\right)}+ \left({Y}_{max2}-{Y}_{max1}\right)\cdot {e}^{-e\cdot {\mu}_{max2}\cdot \left({\lambda}_{2}-t\right)}$ Y (cell⋅mL^−1^), cell concentration at time t; Y_max1_ and Y_max2_ (cell⋅mL^−1^) asymptotic cell concentrations of phase 1 and phase 2 t (d), time λ_1_ and λ_2_ (d), lag times of phase 1 and phase 2; μ_max1_ and μ_max2_ (d^−1^) specific growth rates during phase 1 and phase 2	(2)	(Palafox-Sola et al. 2023)
Biomass—Logistic $Y= {Y}_{max}\cdot \frac{1}{1+{exp}^{{\mu}_{max}\cdot \left({\lambda }{\prime}- t\right)}}$ Y (cell⋅mL^−1^), cell concentration at time t; Y_max_ (cell⋅mL^−1^), asymptotic cell concentration; t (d), time λ’, defined as the time to reach the inflexion point; μ_max_ (d^−1^), maximum specific growth rate	(3)	(Carbonell Sorí et al. 2025; Kothari et al. 2024; López-Pacheco et al. 2019)
Biomass—Verhulst $Y= {Y}_{o}\cdot {Y}_{max}\cdot \frac{{e}^{{\mu}_{max}\cdot t}}{{Y}_{max}- {Y}_{o}+ {Y}_{o}\cdot {e}^{{\mu}_{max}\cdot t}}$ Y (cell⋅mL^−1^), cell concentration at time t; Y_o_ (cell⋅mL^−1^), cell concentration at time 0; Y_max_ (cell⋅mL^−1^), asymptotic cell concentration; t (d), time μ_max_ (d^−1^), maximum specific growth rate	(4)	(Padri et al. 2025, 2022)
Specific growth rate—Exponential $\mu = \frac{\mathrm{l}\mathrm{n}(\frac{{Y}_{1}}{{Y}_{2}})}{{t}_{1}-{t}_{2}}$ Y_1_ and Y_2_ (cell⋅mL^−1^), cell concentrations at two sampling times; t_1_, and t_2_ (d), sampling times; μ (d^−1^), specific growth rate	(5)	(Gao et al. 2018; Gupta and Pawar 2019; Najar-Almanzor et al. 2024; Nasir et al. 2023; Tan et al. 2021; Trovão et al. 2025)
Biomass Productivity – Stationary condition $\frac{\delta Y}{\delta \theta }= {Y}_{max}\cdot \left(-{\theta }^{-2}+ \frac{2}{{\mu}_{max}}\cdot {\theta }^{-3}\right)$ Y (cell⋅mL^−1^), cell concentration at time t; Y_max_ (cell⋅mL^−1^), asymptotic cell concentration; μ_max_ (d^−1^), maximum specific growth rate; θ (d), residence time	(6)	(Ruiz et al. 2013)
Optimal residence time – Stationary condition ${\theta}_{opt}= \frac{2}{{\mu}_{max}}$ μ_max_ (d^−1^), maximum specific growth rate; θ_opt_ (d), residence time to achieve maximum productivity	(7)	(Ruiz et al. 2013)
Specific growth rate—Monod $\mu = {\mu}_{max} \cdot \frac{S}{S+ {K}_{s}}$ K_s_ (mg·L^−1^), substrate saturation constant; S (mg·L^−1^), substrate concentration; μ (d^−1^), specific growth rate; μ_max_ (d^−1^), maximum specific growth rate	(8)	(Das et al. 2022; Palafox-Sola et al. 2023)
Specific growth rate—Andrews $\mu = {\mu}_{max} \cdot \frac{S}{S+ {K}_{s}+\frac{{S}^{2}}{{K}_{is}}}$ ${S}_{opt }= \sqrt{{K}_{s}\cdot {K}_{is}}$ K_is_ (mg·L^−1^), substrate inhibition constant; K_s_ (mg·L^−1^), substrate saturation constant; S (mg·L^−1^), substrate concentration; S_opt_ (mg·L^−1^), optimal substrate concentration μ (d^−1^), specific growth rate; μ_max_ (d^−1^), maximum specific growth rate	(9) (10)	(Blanco et al. 2021)
Population density—Huisman $$\mathrm{A}=\frac{{a}_{H}- {b}_{H}\cdot {k}_{bg}}{{b}_{H}\cdot k}$$ + $$\left[{A}_{o}-\frac{{a}_{H}- {b}_{H}\cdot {k}_{bg}}{{b}_{H}\cdot k}\right]$$$${e}^{-{b}_{H}\cdot t}$$ $${a}_{H}= \frac{{\mu}_{max}}{{z}_{max}} \cdot ln\left(\frac{{ h}_{p}+{I}_{n}}{{ h}_{p}+{I}_{out}}\right)$$ $${b}_{H} = {o}_{r}+{d}_{r}$$ Ao and A, initial and actual optical density of the culture; I_in_ and I_out_ (μE·m^−2^·d^−1^), incoming and outgoing light; a_H_ and b_H_ (d^−1^), increase and decrease rate for microorganism concentration; d_r_ and o_r_ (d^−1^), specific maintenance (death rate) and outflow/dilution; h_p_ (μE·m^−2^·d^−1^), half-saturation of photosynthesis; k_bg_ and k, background turbidity and specific light attenuation; t (d), time; z_max_, mixing depth	(11) (12) (13)	(Huisman et al. 2006)
Biomass—Polynomial quadratic multifactorial $Y= {\alpha}_{0}+ \Sigma {\alpha}_{n}\cdot {X}_{n}+ \Sigma {\alpha}_{nn}\cdot {X}_{n}^{2}+\Sigma {\alpha}_{nm}\cdot {X}_{n}{\cdot X}_{m}$ X_n_ and X_m_ (mg·L^−1^, or °C, or μE·m^−2^·s^−1^, etc..), concentration/ intensity of the variables “n” and “m”; Y (cell⋅mL^−1^), cell concentration at time t; α_o_, offset term; α_n_, linear effect; α_nm_, first-order interaction effect; α_nn_, squared effect for each independent variable	(14)	(Dehghan Banadaki et al. 2024; Dhandayuthapani et al. 2022; Kim et al. 2023; Kumari et al. 2022; Pekkoh et al. 2024; Zhao et al. 2024)
Lipid—Polynomial quadratic multifactor model $L = {\alpha}_{0}+ \Sigma {\alpha}_{n}\cdot {X}_{n}+ \Sigma {\alpha}_{nn}\cdot {X}_{n}^{2}+\Sigma {\alpha}_{nm}\cdot {X}_{n}{\cdot X}_{m}$ L (mg·L^−1^), output concentration (lipid or polyunsaturated fatty acids); X_n_ and X_m_ (mg·L^−1^, etc.), concentration of the variables “n” and “m”; α_o_, offset term; α_n_, linear effect; α_nm_, first-order interaction effect; α_nn_, squared effect for each independent variable	(15)	(Sriket et al. 2024)
Hydrogen—Enhanced Gompertz equation for output production $H={H}_{max}\cdot exp\left[-exp\left[\frac{e\cdot {R}_{max}\cdot \left(l-t\right)}{{P}_{max}}+1\right]\right]$ H (mg⋅g^−1^) and H_max_ (mg⋅g^−1^), actual and maximum output (hydrogen) productivity; P_max_ (mL·g microalgae^−1^), maximum output (hydrogen) production potential; R_max_ (mL·g microalgae^−1^·d^−1^), maximum output (hydrogen) production rate; l (d), pre-treatment duration	(16)	(Ahmad Sobri et al. 2023)
Contaminant removal rate—Monod $\frac{dC}{dt} = {\left(\frac{dC}{dt}\right)}_{max} \cdot \frac{C}{C+ {K}_{s}}$ C (mg·L^−1^), contaminant (COD, TN, TP, etc..) concentration; K_s_ (mg·L^−1^), saturation constant for C; t (d), time	(17)	(Sudhanthiran and Perumalsamy 2024; Das et al. 2022)
Contaminant removal rate—Zero-order $\frac{dC}{dt}={- k}_{0 order}$ C (mg·L^−1^), contaminant (COD, TN, TP, etc..) concentration; k_0 order_ (mg·L^−1^·d^−1^), first-order rate constant; t (d), time	(18)	(Bedane and Asfaw 2023; Padri et al. 2025; Palafox-Sola et al. 2023)
Contaminant removal rate—First-order $\frac{dC}{dt}={- k}_{1st order}\cdot C$;${t}_\frac{1}{2}=\frac{ln2}{{- K}_{1st order}}$ C (mg·L^−1^), contaminant (COD, TN, TP, etc..) concentration; k_1st order_ (d^−1^), first-order rate constant; t (d), time; t_1/2_ (d), time to decrease “C” by 50%	(19)	(Aranda-Vega et al. 2024; Bhatt et al. 2024; Kothari et al. 2024; Širić et al. 2023)
Contaminant removal rate—Polynomial quadratic multifactorial $\frac{dC}{dt} = {\alpha}_{0}+ \Sigma {\alpha}_{n}\cdot {X}_{n}+ \Sigma {\alpha}_{nn}\cdot {X}_{n}^{2}+\Sigma {\alpha}_{nm}\cdot {X}_{n}{\cdot X}_{m}$ C (mg·L^−1^), contaminant (COD, TN, TP, etc..) concentration; X_n_ and X_m_ (mg·L^−1^, μE·m^−2^·s^−1^, etc.), content/intensity of the variables “n” and “m”; t (d), time; t_1/2_ (d), time to decrease “C” by 50% α_o_, offset term; α_n_, linear effect; α_nm_, first-order interaction effect; α_nn_, squared effect for each independent variable	(20)	(Zhao et al. 2024)
Biomass of “i” and “j”—Simplified Lotka-Volterra $\frac{{dY_{i} }}{{{\mathrm{dt}}}}{ = }\frac{{\mu_{i} \cdot Y_{i} \left( {K_{1} - \beta_{j} \cdot Y_{j} } \right)}}{{K_{1} }}$; $\frac{{dY_{j} }}{{{\mathrm{dt}}}}{ = }\frac{{\mu_{j} \cdot Y_{j} \left( {K_{2} - \beta_{i} \cdot Y_{i} } \right)}}{{K_{2} }}$ Microorganism”i” inhibits microorganism”j” if: β_j_ < K_1_/K_2_ and β_i_ > K_2_/K_1_, and vice-versa Y_i_ and Y_j_ (cell⋅mL^−1^), concentrations of the microorganisms “i” and “j”, respectively; *μ *_i_ and *μ *_j_ (d^−1^), maximum specific growth rates for microorganisms “i” and “j”, respectively, in monoculture *K*_1_ and *K*_2_ (cell·mL^−1^), carrying capacities of microorganisms “i” and “j”, respectively, in co-culture; β_i_, is the competition coefficient of “j”against “i”; β_j_, is the competition coefficient of “i” against “j”	(21)	(Ding et al. 2024)
Biomass of “i” and “j”- Modified Lotka-Volterra $\frac{d{Y}_{i}}{dt}= {\mu}_{i}\cdot {Y}_{i}+ {\beta}_{ii }\cdot {Y}_{i}^{2}+{\beta}_{ij}\cdot {Y}_{i}\cdot {Y}_{j}; \frac{d{Y}_{j}}{dt}= {\mu}_{j}\cdot {Y}_{j}+ {\beta}_{jj }\cdot {Y}_{j}^{2}+{\beta}_{ji}\cdot {Y}_{j}\cdot {Y}_{i}$ Synergism occurs if β_ij_ > 0 Antagonism occurs if β_ij_ < 0 Y_i_ and Y_j_ (cell⋅mL^−1^), concentrations of the microorganisms “i” and “j”, respectively; *μ *_i_ and *μ *_j_ (d^−1^), maximum specific growth rates for microorganisms “i” and “j”, respectively, in monoculture β_ii_ and β_jj_ (mL·cell^−1^·d^−1^), parameters that describe intraspecific interactions; β_ij_ and β_ji_ (mL·cell^−1^·d^−1^), parameters that describe interspecific interactions	(22)	(Palafox-Sola et al. 2023; Roy et al. 2023)

### Application of the Gompertz model

The modified Gompertz model is particularly well suited for the preliminary assessment and optimization of *Chlorella* growth at the laboratory scale in batch cultures, especially in effluents where complex C sources, high turbidity that limits light availability, or the presence of toxic compounds result in extended lag phases and/or reduced growth rates. Effluents from tortilla processing, for example, exhibited longer lag phases and lower growth rates compared with synthetic media such as Bold Basal Medium (BBM) or Blue-Green 11 (BG-11) (Najar-Almanzor et al. 2024). Prolonged lag phases, reaching up to 14.2 d, were also reported for algal growth in slaughterhouse wastewater (Pérez-Guzmán et al. 2024; Kothari et al. 2024). In a highly complex substrate such as anaerobically digested swine wastewater, the emergence of biphasic growth behavior was successfully captured using the double Gompertz equation, which revealed sequential substrate utilization, with an initial phase supported by readily assimilable compounds followed by a second phase driven by more recalcitrant nutrients (Palafox-Sola et al. 2023) (Table 2). These findings underscore the usefulness of Gompertz-type models not only for fitting experimental data but also for interpreting algal metabolic adaptation to different substrates. From an operational perspective, the Gompertz model can serve as an optimization tool. For the treatment of sugarcane vinasse (Serejo et al. 2021) and mushroom factory effluents (Širić et al. 2023), this tool has been applied to identify optimal dilution conditions. For the digested gelatin effluent, it was used to determine the optimal ratio between wastewater and synthetic BBI. Moreover, its application has proven informative during scale-up: for digested gelatin wastewater, comparable μ_max_ values (0.343 d^−1^) have been reported when transitioning from laboratory-scale cultures to a bubbled photobioreactor, although increases in lag phase (1.77 d), have been observed. The longer lag phase observed on the larger scale was attributed to the lower efficiency of mixing and mass transfer in the pneumatic agitation system of the bubble-column photobioreactor compared with the laboratory-scale shaker, which resulted in a longer adaptation period for the microorganisms (Blanco et al. 2021). However, the specific mechanism underlying this phenomenon—such as, for instance, a slow rate of enzymatic hydrolysis of the C source caused by the inefficient diffusion of inhibitors, including products or substrates—was not identified. Nevertheless, this finding highlights a critical limitation: models developed at smaller scales may not adequately account for the hydrodynamic and mass-transfer constraints encountered in larger-scale systems..

**Table 2:** Kinetic studies on the cultivation of *Chlorella* spp. in mono and co-culture in effluents of the food and beverage industry

Species	Effluent and quality	Cultivation conditions	Biomass/bioproducts’ yield	Pollutants’ removal	Reference
*Chlorella* sp.	Effluent of tortilla processing COD (mg·L^−1^): 32,880 TN (mg·L^−1^): 170 TP (mg·L^−1^): 1113	Lab scale, batch mode Pretreatment: filtration (screen 0.7 mm) and pH adjustment at 7.0 Light (lx): 3000 Light/dark cycle: n.s Duration (d): 14 Temperature (°C): 28	Parameters for modified Gompertz model: λ (d): 0 μ_max_ (d^−1^): 0.665 Output: Biomass (g·L^−1^): 0.97 Protein (%):16.93 Pigments (mg·g^−1^): 1.62 Phenolics (mg·g^−1^): 2.87	Removal efficiency: COD (%): 85 TN (%): 100 TP (%): 100	(Najar-Almanzor et al. 2024)
*Chlorella vulgaris*	Effluent of slaughterhouse COD (mg·L^−1^): 3243 NO_3_^−^ (mg·L^−1^): 298 PO_4_^3−^ (mg·L^−1^): 26 pH: 10.7	Lab scale, batch mode Pretreatment: dilution 50% Light (W·m^−2^): 10 Light/dark cycle (h/h): 12/12 Duration (d): 15 Temperature (°C): 30	Parameters for modified Gompertz model: λ (d): 5.94 μ_max_ (d^−1^): 0.315 Parameters for logistic model: λ’ (d): 7.61 μ_max_ (d^−1^): 0.578 Output: Biomass (g·L^−1^): 0.39 Lipid (%): 30.7	Parameters for first-order removal kinetics: k_1st order_ BOD (d^−1^): 0.0411 k_1st order_ COD (d^−1^): 0.0429 k_1st order_ NO_3_^−^ (d^−1^): 0.0508 k_1st order_ PO_4_^3−^ (d^−1^): 0.0450 Removal efficiency: BOD (%): 32 – 75 COD (%): 32 – 77 NO_3_^−^ (%): 33 – 82 PO_4_^3−^ (%): 29 – 78	(Kothari et al. 2024)
*Chlorella vulgaris*	Effluent of poultry slaughterhouse COD (mg·L^−1^): 39,950 TN (mg·L^−1^): 1900 TP (mg·L^−1^): 0.00049 pH: 6.04	Lab scale, batch mode Pretreatment: addition of ZnCl_2_, MnCl_2_, NaMoO_4_ and CuSO_4_ (MN) Air supply (cm^3^·min^−1^): 1500 Light (μE ·m^−2^·s^−1^): 35 Light/dark cycle (h/h): 12/12 Duration (d): 42 Temperature (°C): 25	Parameters for modified Gompertz model: λ (d): 20.5 (control); 8.47 (MN) μ_max_ (d^−1^): 0.225 (control); 0.449 (MN) Output: Biomass (g·L^−1^): 61.53	Removal efficiency: COD (%): 99.14 TN (%): 99.33	(Pérez-Guzmán et al. 2024)
*Chlorella vulgaris*	Effluent of swine processing Initial composition: COD (mg·L^−1^): 808.00 TN (mg·L^−1^): 808.08 TP (mg·L^−1^): 70.96 pH: 7.87	Lab scale, batch mode Pretreatment: anaerobic digestion Light (natural, μE ·m^−2^·s^−1^): 97 at 12:00 pm and 3 at 6:00 pm Light/dark cycle (h/h): 13/11 Duration (d): 30 Temperature (°C): 30	Parameters for modified double Gompertz model: λ_1_ (d): 0.71 d, λ_2_ (d): 9.69 d μ_1_ (d^−1^): 0.63, μ_2_ (d^−1^): 0.89 Parameters for Monod model: Ks COD (mg·L^−1^) 2.27 Ks TN (mg·L^−1^): 2.28 Ks TP (mg·L^−1^) 2.31 Output: Biomass (g·L^−1^) 1.51 Lipid (%): 6.3	Parameters for zero-order removal kinetics: k_0 order_ COD (mg·L^−1^·d^−1^): 89.8 k_0 order_ TN (mg·L^−1^·d^−1^): 55.0 k_0 order_ TP (mg·L^−1^·d^−1^): 9.72 Removal efficiency: COD (%): 46 TN (%): 45 TP (%): 67	(Palafox-Sola et al. 2023)
*Chlorella vulgaris*	Effluent of sugarcane filtered (FT) or not filtered (UFT) Initial composition: COD (mg·L^−1^): 2700 (FT), 3200 (UFT) NH_4_^+^ (mg·L^−1^): 64 (FT), 61(UFT) TN (mg·L^−1^): 110 (FT), 148 (UFT) PO_4_^3−^ (mg·L^−1^): 191 (FT), 216 (UFT) pH: 7.6 (FT and UFT)	Lab scale, batch mode Pretreatment: filtration, anaerobic digestion, Light (μE·m^−2^·s^−1^): 47 Light/dark cycle (h/h): 16/8 Duration (d): 35 (16 to reach the steady state) Temperature (°C): 30	Parameters for modified Gompertz model: λ (d): n.r μ_max_ (d^−1^): 0.88 (FT), 0.60 (UFT) Output: Biomass (g·L^−1^): 1.3 (FT) and 2.7 (UFT) Lipid (%): 9.2 (FT) and 9.8 (UFT)		(Serejo et al. 2021)
*Chlorella vulgaris*	Effluent gelatin processing supplemented with BBM Initial composition: NH_4_^+^( mg·L^−1^): 281.9 NO_2_^−^ (mg·L^−1^): 10.44 NO_3_^−^ (mg·L^−1^): 0.22 N_org_ (mg·L^−1^): 78.08 TP (mg·L^−1^): 2.05 pH: 7.75	Lab scale and photobioreactor, batch mode Pretreatment: anaerobic digestion, dilution Light (lx): 4600 Light/dark cycle (h/h): 24/0 Duration (d): 7 Temperature (°C): 26	Parameters for modified Gompertz model: λ (d): 0 – 0.97 (lab scale); 1.77 (photobioreactor) μ_max_ (d^−1^): 0 – 0.364 (lab scale); 0.343 (photobioreactor) Parameters for Andrews model: K_opt_ NH_4_^+^ (mg ·L^−1^): 37.59 Output: Biomass (g·L^−1^): 1.83 Lipid (%): 12.45		(Blanco et al. 2021)
*Chlorella vulgaris*	Effluent of fish processing Initial composition: BOD (mg·L^−1^): 2941 COD (mg·L^−1^): 5588 TN (mg·L^−1^): 169.2 PO_4_^3−^ (mg·L^−1^): 78.2 pH: 6.4	Lab scale, batch mode Light (μE ·m^−2^·s^−1^): 48 Light/dark cycle (h/h): 12/12 Duration (d): 13 Temperature (°C): 24.5	Parameters for logistic model: λ’ (d): 5.98, μ_max_ (d^−1^): 0.51 Output: Biomass (g·L^−1^): 1.09	Removal efficiency: TN (%): 98.11 PO_4_^3−^ (%): 98.45	(Carbonell Sorí et al. 2025)
*Chlorella vulgaris*	Effluent of maize processing (N, nejayote) and swine processing (S) Initial composition: COD (mg·L^−1^): 10,933 (S) and 9153 (N) TN (mg·L^−1^): 163 (S) and (121 (N) TP (mg·L^−1^): 147 (S) and 41 (N) pH: 6.83 (S) and 9.80 (N)	Lab scale, batch mode Pretreatment: dilution and mixing of N and S at different ratios Air supply (L·m^−2^·min^−1^): 1.5 Light: n.s Light/dark cycle (h/h): 12/12 Duration (d): 20 Temperature (°C): 21	Parameters for logistic model: Best combination: 20% S in water λ’ (d): 2.19 μ_max_ (d^−1^): 0.57 Output: Biomass (g·L^−1^): 1.7	Removal efficiency: COD (%): 96 TN (%): 91 TP (%): 85	(López-Pacheco et al. 2019)
*Chlorella sorokiniana* (P21) and natural communities of algae (P2 and P3)	Effluent of cassava processing Initial composition: COD (mg·L^−1^): 205 NO_3_^−^( mg·L^−1^): 16.43 TN (mg·L^−1^): 54.1 PO_4_^3−^ (mg·L^−1^): 25.53 TP (mg·L^−1^): 37.27	Lab scale, batch mode Pretreatment: anaerobic digestion, filtration (1 mm screen) Light (μE ·m^−2^·s^−1^): 81 Light/dark cycle (h/h): 24/0 Duration (d): 7 Temperature (°C): 25	Parameters for the Verhulst model: μ_max_ (d^−1^) = 0.5 (P21), 0.65 (P2) and 0.69 (P3) Output: Biomass (g·L^−1^): 1.47 (P21) 1.19 (P2) and 0.901 (P3)	Removal efficiency: COD (%): ≈ 30 (P21), ≈ 40 (P2), ≈ 20 (P3) NO_3_^−^ (%): ≈ 80 (P21), ≈ 70 (P2), ≈ 70 (P3) PO_4_^3−^ (%): ≈ 80 (P21), ≈ 80 (P2), ≈ 70 (P3)	(Padri et al. 2022)
*Chlorella sorokiniana (Cs)* and *Streptomyces thermocarboxydus (St)* co-culture	Effluent of cassava processing unsterilized (US) and sterilized (S) Initial composition: BOD (mg·L^−1^): 75 COD (mg·L^−1^): 205 NH_4_^+^(mg·L^−1^): 31.24 NO_2_^−^ (mg·L^−1^): 0.08 NO_3_^−^ (mg·L^−1^): 16.43 TN (mg·L^−1^): 54.1 PO_4_^3−^ (mg·L^−1^): 23.53 TP (mg·L^−1^): 37.26 pH: 7.6	Lab scale, batch and semi-continuous mode Pretreatment: anaerobic digestion, sterilization and non-sterilization Air supply (L·min^−1^): 1.5–2 Light (μE ·m^−2^·s^−1^): 81 Light/dark cycle (h/h): 12/12 Duration (d): 16 (batch mode), 72 (semicontinuous mode) Temperature (°C): 28	Parameters for the Verhulst model: (batch system: US was better than S) μ_max_ (d^−1^): 0.35 (Cs, US) and 0.25 (Cs + St, US) Output: (semi-continuous system with θ_max_ (d) = 5, US) Biomass productivity (g·L^−1^·d^−1^): 0.13 (Cs, US) and (Cs + St, US) 0.14	Parameters for zero-order removal kinetics: (Semi-continuous system with θ_max_ (d) = 5, US) k_0 order_ COD (mg·L^−1^·d^−1^): 10 (Cs, US) and 14 (Cs + St, US) k_0 order_ TN (mg·L^−1^·d^−1^): 1.0 and 1.4 k_0 order_ TP (mg·L^−1^·d^−1^): 1.1 (Cs, US and Cs + St, US)	(Padri et al. 2025)
*Chlorella* sp.	Effluent of cheese processing Initial composition: COD (mg·L^−1^): 77,100 TN (mg·L^−1^): 340 TP (mg·L^−1^): 2810	Lab scale, batch mode Pretreatment: pH adjustment at 7.0, pasteurization at 80 °C for 30 s Light (lx): 3000 Light/dark cycle: n.s Duration (d): 14 Temperature (°C): 28	Specific growth rate: μ (d^−1^): 0.115 Output: Biomass (g·L^−1^): 0.89 Protein (%): 14.7 Pigments (mg·g^−1^): 0.22 Phenolics (mg·g^−1^): 0.87	Removal efficiency: COD (%): 3.14 TN (%): 3.70 TP (%): 17.05	(Najar-Almanzor et al. 2024)
*Chlorella* sp.	Effluent of tequila processing Initial composition: COD (mg·L^−1^): 132,120 TN (mg·L^−1^): 150 TP (mg·L^−1^): 1713	Lab scale, batch mode Pretreatment: filtration and pH adjustment at 7.0 Light (lx): 3000 Light/dark cycle: n.s Duration (d): 14 Temperature (°C): 28	Specific growth rate: μ (d^−1^): 0.129 Output: Biomass (g·L^−1^): 1.23 Protein (%): 17.93 Pigments (mg·g^−1^): 0.36 Phenolics (mg·g^−1^): 1.02	Removal efficiency: COD (%): 70.53 TN (%): 66.18, TP (%): 1.52	(Najar-Almanzor et al. 2024)
*Chlorella* sp.	Effluent of shrimp aquaculture in marine water (MW) and fresh water (FW) Initial composition: NH_4_^+^(mg·L^−1^): 2.80 (MW) and 3.6 (FW) NO_2_^−^ (mg·L^−1^): 1.5 (MW) and 1.75 (FW) PO_4_^3−^ (mg·L^−1^): 4.1 (MW and FW)	Lab scale, batch mode Light: n.s Light/dark cycle: n.s Duration (d):10 Temperature: n.s	Specific growth rate: μ (d^−1^): 0.18 (MW); 0.15 d^−1^ (FW)	Removal efficiency: NH_4_^+^ (%): 96.77 (MW) and 90.10 (FW) % NO_2_^−^ (%): 82.07 (MW) and 87.09 (FW) PO_4_^3−^ (%): 75.96 (MW) and 95.6 (FW) %	(Nasir et al. 2023)
*Chlorella* sp.	Effluent of seafood processing (SPW) Initial composition: COD (mg·L^−1^): 1220 (SPW), 295 (ASPW), 553 (DSPW) NO_2_^−^ (mg·L^−1^): 0.73 (SPW), 3.21 (ASPW), 0.29 (DSPW) NO_3_^−^ (mg·L^−1^): 6.99 (SPW), 47.84 (ASPW), 3.42 (DSPW) TN (mg·L^−1^): 121(SPW), 95 (ASPW), 45 (DSPW) pH: 7.92 (SPW), 7.14 (ASPW), 7.53 (DSPW)	Lab scale, batch mode Pretreatment: aeration (ASPW) or dilution (DSPW) Light (μE·m^−2^·s^−1^): 135 Light/dark cycle (h/h): 24/0 Duration (d): 45 Temperature (°C): 25	Specific growth rate: μ (d^−1^): 0.040 (SPW), 0.156 (ASPW), 0.091 (DSPW) Output: Biomass (g·L^−1^): 0.896 (SPW), 1.243 (ASPW), 0.800 (DSPW) Lipid (%): 25.4 – 28.7 (no significant difference among SPW, ASPW, DSPW)	Removal efficiency: TN (%): 96.6 (SPW), 94.5 (ASPW), 89.8 (DSPW) TP (%): 68.4 (SPW), 74.8 (ASPW), 77.6 (DSPW)	(Gao et al. 2018)
*Chlorella vulgaris* yellow mutant (chlorophyll-deficient strain)	Corn molasses Initial composition: C (%): 35 (CM), 16 (FW 1), 16 (FW 2) H (%): 6.61 (CM), 5.57 (FW 1), 7.25 (FW 2) N (%): 0.31 (CM), 23 (FW 1), 30 (FW 2) P (mM): 4.9 (CM), 369 (FW 1), 1377 (FW 2) pH: 6.5	Lab scale and bioreactor, fed-batch mode to stabilize ammonia content and pH Mixing of FW1, FW2 and CM at ratios: 0.8: 0.5: 3.5 Light/dark cycle: n.s Duration (d): 7 Temperature (°C): 30	Specific growth rate: μ_max_ (d^−1^): 1.09 (lab scale), 1.0 (bioreactor) Output: Biomass (g·L^−1^): 9.06 (lab scale), 154 (bioreactor) Protein (%): 50 (lab scale), 30 (bioreactor)		(Trovão et al. 2025)
*Chlorella pyrenoidosa*	Effluent of starch (ADSW) and alcohol (AW) processing COD (mg·L^−1^): 45,683 (AW), 792 (ADS W) NH_4_^+^ (mg·L^−1^): 214 (AW), 273 (ADSW) TN (mg·L^−1^): 725 (AW), 307 (ADSW) PO_4_^3−^ (mg·L^−1^): 19 (AW), 27 (ADSW) TP (mg·L^−1^): 64 (AW), 37 (ADSW) pH: 3.2 – 4.5 (AW), 7.2 – 7.3 (ADSW)	Reactor for outdoor cultivation, batch mode Anaerobic digestion for ADSW and acidification for alcohol wastewater (AW); filtration Light: n.s Light/dark cycle: n.s Duration (d): 12 Temperature (°C): 30 – 32	Specific growth rate: (at the optimal AW: ADSW ratio = 1:15) μ_max_ (d^−1^): 0.98 Output: (at the optimal AW: ADSW ratio = 1:15) Biomass (g·L^−1^): 2.76 Lipid (%): 19	Removal efficiency: (at the optimal AW: ADSW ratio = 1:15) COD (%): 100 NH_4_^+^ (%): 87.71 TP (%): 87	(Tan et al. 2018)
*Chlorella pyrenoidosa*	Effluent of soybean processing Initial composition COD (mg·L^−1^): 869.8 – 1191.6 NH_4_^+^ (mg·L^−1^): 232.7 – 260.3 TN (mg·L^−1^): 285.7 – 302.9 PO_4_^3−^(mg·L-1): 14.5 – 18.2 pH: 7.0 – 7.3	Reactor for outdoor cultivation, batch mode (spring, summer, fall) Average daily light (lx): 3.72·10^4^ (SP, spring), 7.93·10^4^ (SU, summer), 5.07·10^4^ (FA, fall) Duration (d): 14 (SP, F), 8 (F) Average temperature (°C): 19.2 (SP), 31.8 (SU), 22.5 (FA)	Specific growth rate: μ_max_ (d^−1^): 0.73 (SP), 0.85 (FA), 1.59 (SU) Output: Biomass (g·L^−1^): 1.83—2.10 Protein (%): 56.8 (SP), 60.0 (SU), 56.6 (FA) Lipid (%): 15.3 (SP), 8.1 (SU), 12.4 (FA) Carbohydrate (%): 20.2 (SP), 23.5 (SU), 18.5 (FA)	Removal efficiency: COD (%): 40 – 55 NH_4_^+^(%): 80—90 TN (%) ≈ 60—80 TP (%): ≈ 60—70	(Tan et al. 2021)
*Chlorella* sp.	Effluent of hydroponic lettuce cultivation added with glucose (GLU) and indole−3-acetic acid (IIA) Initial composition: COD (mg·L^−1^): 88 NH_4_^+^(mg·L^−1^): 3.7 NO_3_^−^ (mg·L^−1^): 93 PO_4_^3−^ (mg·L^−1^): 235.5	Lab scale, batch mode Light: none Light/dark cycle (h/h): 0/24 Duration (d): 6 Temperature (°C): 25	Parameters for polynomial quadratic model: X_1_ = GLU concentration, X_2_ = IIA concentration α_o_: − 2.14·10^–2^; α_1_: 0.13; α_2_: 0.11; α_11_: − 6.11·10^–3^; α_22_: − 5.46·10^–3^; α_12_: 0 Output: Biomass (g·L^−1^): 1.32 Lipid (%): 6.3 (yield_max_ for X_1_ =10.89 mg·L^−1^ and X_2_ =10.15 mg·L^−1^)	Removal efficiency: COD (%): 92 NH_4_^+^ (%): 100 NO_3_ (%): 96.77 PO_4_^3−^ (%) 97.03	(Pekkoh et al. 2024)
*Chlorella vulgaris*	Effluent of hydroponic lettuce cultivation added with glucose (GLU) and indole−3-acetic acid (IIA) Initial composition: COD (mg·L^−1^): 14,490.7 NH_4_^+^(mg·L^−1^): 120 NO_3_^−^ (mg·L^−1^): 145 PO_4_^3−^ (mg·L^−1^): 256	Lab scale, batch mode Light: none Light/dark cycle (h/h): 0/24 Duration (d): 6 Temperature (°C): 25	Parameters for polynomial quadratic model: X_1_ = GLU concentration, X_2_ = IIA concentration For biomass: α_o_: 1.33; α_1_: 0.17; α_2_: − 0.084; α_11_: − 10^−2^; α_22_:-0.53·10^–3^; α_12_: − 5.4·10^–3^ For lipid content: α_o_: 4.03; α_1_: 0.12; α_2_:0.32; α_11_: − 2.7·10^–3^; α_22_: 0.57·10^–3^; α_12_: 7.8·10^–3^ Output: Biomass (g·L^−1^): 2.8 Lipid (%): 8.29 (yield_max_ for X_1_ = 12.2 mg·L^−1^ and X_2_ = 15 mg·L^−1^)	Removal efficiency: COD (%): 88.74 NH_4_^+^ (%): 81.21 NO_3_ (%): 93.79 PO_4_^3−^ (%) 89.94	(Sriket et al. 2024)
*Chlorella* sp.	Effluent of soy sauce (SSW) and defective soy sauce (DSS) Initial composition: COD (mg·L^−1^): 6700 (SWW) and 790,000 (DSS) NH_4_^+^(mg·L^−1^): 15 (SSW) and 1700 (DSS) NO_2_^−^ (mg·L^−1^): 0 (SSW) and 36,869 (DSS) NO_3_^−^ (mg·L^−1^): 3.3 (SSW) and 0 (DSS) PO_4_^3−^ (mg·L^−1^): 3.3 (SSW) and 0 (DSS)	Lab scale, batch mode Light (μE·m^−2^·s^−1^): 20—800 Ligh/dark cycle: n.s Duration (d):10 Temperature (°C): 20—40	Parameters for polynomial quadratic model X_1_ = temperature, X_2_ = light intensity α_o_: − 3.98; α_1_: 0.37; α_2_: 0.0017; α_11_: − 0.0065; α_22_: − 0.000 (SSW) α_o_: − 3.65; α_1_: 0.28; α_2_: 0.0007; α_22_: − 0.0042; α_12_: − 0.000035 (DSS) Output: Biomass (g·L^−1^): 1.05 (SSW, yield_max_ for X_1_ = 25 °C and X_2_ = 491 μE·m^2^s^−1^); 1.37 (DSS, yield_max_ for X_1_ = 34 – 35 °C and X_2_ = 100 – 300 μE·m^−2^·s^−1^)		(Kim et al. 2023)
*Chlorella vulgaris*	Effluent of dairy products’ processing Initial composition: BOD (mg·L^−1^): 1450 COD (mg·L^−1^): 2510 TN (mg·L^−1^): 162 TP (mg·L^−1^): 128 pH: 7.52	Lab scale, batch mode Pretreatment: dilution (0—100%) Light (lx): 2000—4000 Light/dark cycle (h/h): 18/6 Duration (d): 16 Temperature (°C): 20 – 30	Parameters for polynomial quadratic model: X_1_ = substrate dilution, X_2_ = temperature, X_3_ =. light intensity α_o_: 1.68; α_1_: 0.33; α_2_: 0.01; α_3_:0.07; α_11_: − 0.60; α_22_: − 0.12; α_33_: − 0.03; α_12_: − 0.02; α_13_: 0.01; α_23_: 0.01 Output: Biomass (g·L^−1^): 1.757 (yield_max_ for X_1_ = 57.72%, X_2_ = 24.16 °C, X_3_ = 3874.51 lx)	Parameters for polynomial quadratic model: X_1_ = substrate dilution, X_2_ = temperature, X_3_ =. light intensity α_o_: 83.13; α_1_: 16.2; α_2_: 0.97; α_3_: 3.96; α_11_: − 30.12; α_22_: − 6.31; α_33_: − 0.75; α_12_: − 1.09; α_13_: 0.43; α_23_: 0.38 Removal efficiency: COD (%): 87.5 (yield_max_ for X_1_ = 57.72%, X_2_ = 24.16 °C, X_3_ = 3874.51 lx)	(Kumari et al. 2022)
*Chlorella* sp.	Effluent of marine aquaculture Initial composition: COD (mg·L^−1^): 47.39 NO_3_^−^ (mg·L^−1^): 44.14 TP (mg·L^−1^): 13.47	Lab scale, batch mode Pretreatment: autotrophic denitrification and nitrification integrated constructed wetland Light (μE ·m^−2^·s^−1^): 50—200 Light/dark cycle: 0/24 – 24/0 Duration (d): 8 Temperature (°C): 25	Parameters for the polynomial quadratic model X_1_ = OD_680_; X_2_ = light intensity; X_3_ = photoperiod α_0_ =56.59; α_1_= 2.51; α_2_ = 8.61; α_3_ = 2.71; α_12_ = 4.96; α_13_ = 0.1563; α_23_ = − 3.59; α_11_ = − 12.80; α_22_ = − 14.03; α_22_ = − 21.8 Output Biomass (mg·L^−1^·d^−1^): 436 (yield_max_ for X_1_ = 0.394 OD_680_, X_2_ = 161 μE ·m^−2^·s^−1^, X_3_ = 16/18)	Parameters for the polynomial quadratic model: X_1_ = OD_680_; X_2_ = light intensity; X_3_ = photoperiod For TN: α_0_ = 96.21; α_1_ = 6.37; α_2_ = 15.32; α_3_ = 16.45; α_12_= 7.97; α_13_ = − 4.845; α_23_ = 2.43; α_11_ = − 20.83; α_22_ = − 17.49; α_22_ = − 27.61 For TP: α_0_ = 74.03; α_1_ = 6.49; α_2_ = 13.14; α_3_ = 10.45; α_12_= 10.01; α_13_ = − 0.8685; α_23_ = − 1.71; α_11_ = − 21.01; α_22_ = − 17.12; α_22_ = − 20.50	(Zhao et al. 2024)
*Chlorella sorokiniana*	Effluent of dairy product processing Initial composition: COD (mg·L^−1^): 1593 TN (mg·L^−1^): 63.21 TP (mg·L^−1^): 35.15	Lab scale, batch mode Light (μE ·m^−2^·s^−1^): 30 − 100 Light/dark cycle (h/h): 12/12 Duration (d): 10 Temperature (°C): 25 – 45 Agitation: 100 – 200 rpm	Parameters for the polynomial model: X_1_ = light intensity, X_2_ = pH, X_3_ = temperature, X_4_ = agitation speed α_o_: 45.98; α_1_: 0.66; α_2_: 0.77; α_3_:1.06; α_4_: 0.22; α_11_: 0.063; α_22_: − 0.69; α_33_: 94; α_44_: 2.063; α_12_: 0.13; α_13_: − 16.0; α_14_: − 18.0; α_23_: − 22.0; α_24_: − 0.62; α_34_: 2.31 Output: Biomass (g·L^−1^): 16.35 (yield_max_ for X_1_ = 65 μE ·m^−2^·s^−1^, X_2_ = 7.0, X_3_ = 35 °C, X_4_ = 150 rpm) Carbohydrate (%): 27	Removal efficiency: COD (%): 94.8	(Dhandayuthapani et al. 2022)
*Chlorella vulgaris*	Effluent of poultry slaughterhouse Initial composition: BOD (mg·L^−1^): 120 COD (mg·L^−1^): 300 TN (mg·L^−1^): 600 TP (mg·L^−1^): 60 pH: 8.0	Lab scale, batch mode Light (μE ·m^−2^·s^−1^): 65 Light/dark cycle (h/h): 8/16—0/24 Duration (d): 5–15 Temperature (°C): 25	Parameters for the polynomial quadratic model X_1_ = total nitrogen; X_2_ = total phosphorous; X_3_ = culture duration; X_4_ = photoperiod α_0_ = 84.33; α_1_= 3.94; α_2_ = 2.71; α_3_ = 13.62; α_4_ = 3.33; α_12_ = − 3.22; α_13_ =− 2.64; α_14_ = 2.40; α_34_ = − 3.21; α_22_ = − 1.90; α_22_ = − 3.09; α_44_ = − 6.77 Output: Biomass increase (%): 94.89 (yield_max_ for X_1_ = 425 mg·L^−1^, X_2_ = 65 mg·L^−1^, X_3_ =16 h, and X_4_ = 15 d)	Parameters for the polynomial quadratic model X_1_ = total nitrogen; X_2_ = total phosphorous; X_3_ = culture duration; X_4_ = photoperiod COD removal: α_0_ = 76.54; α_1_= 4.42; α_2_ = 4.08; α_3_ = 4.68; α_4_ = 4.66; α_12_ = − 4.09; α_13_ = − 6.48; α_14_ = − 4.86; α_24_ = 3.45; α_34_ = − 6.25; α_22_ = − 5.08; α_22_ = 0.67; α_33_ = − 1.37; α_44_ = 5.62 Removal efficiency: COD (%): 90 (max for X_1_ = 425 mg·L^−1^, X_2_ = 65 mg·L^−1^, X_3_ = 24 h, and X_4_ = 10 d)	(Dehghan Banadaki et al. 2024)
*Chlorella vulgaris* sp.	Effluent of palm kernel expeller Initial composition: n.d	Lab scale, batch mode Heat treatment for 0—10 h at 90 °C Light: none Light/dark cycle (h/h): 0/24 Duration (d): 6 Temperature: n.s	Parameters for the enhanced Gompertz model P_max_ (mL H_2_·g _microalgae_^−1^): 387.9 R _max_ (mL H_2_·g _microalgae_^−1^_·_d^−1^): 140 l (h): 1		(Ahmad Sobri et al. 2023)
*Chlorella pyrenoidosa*	Effluent of dairy products’ processing Initial composition: COD (mg·L^−1^): 720 NH_4_^+^ (mg·L^−1^): 55 NO_3_^−^ (mg·L^−1^): 22 TP (mg·L^−1^): 18 pH: 7.0	Lab scale, batch mode Pretreatment: bacterial digestion Light: natural Light/dark cycle: n.s Duration (d): 8 Temperature: n.s	Output: Biomass (g·L^−1^): 0.84	Parameters for Monod model: Ks COD (mg·L^−1^): 69.43 Ks NH_4_^+^ (mg·L^−1^): 19.12 Ks NO_3_^−^ (mg·L^−1^): 16.21 Ks PO_4_^3−^ (mg·L^−1^): 5.41 Removal efficiency: COD (%): 95 NO_3_^−^ (%): 99.67 NH_4_^+^ (%): 95 PO_4_^3−^ (%): 90.25	(Das et al. 2022)
*Chlorella vulgaris*	Effluent of dairy products’ processing Initial composition: COD (mg·L^−1^): 1290 TN (mg·L^−1^): 128 TP (mg·L^−1^): 38 pH: 7.97	Lab scale, batch mode Pretreatment: dilution (25, 50, 75%) Light (lx): 3500 Light/dark cycle: n.s Duration (d): 17 Temperature (°C): n.s	Output: Biomass (g·L^−1^): 2.43	Parameters for Monod model: Ks TN (mg·L^−1^): 198.77 Ks TP (mg·L^−1^): 668.9 Removal efficiency: COD (%): 81.48 TN (%): 87.7 TP (%): 93.5	(Sudhanthiranand Perumalsamy 2024)
*Chlorella vulgaris*	Effluent of salmon aquaculture Initial composition: NO_3_^−^ (mg·L^−1^): 291 (C) and 160 (P) PO_4_^3−^ (mg·L^−1^): 1.6 (C and P) NO_3_^−^ in the medium (mg·L^−1^): 3172 (C) and 494 (P) PO_4_^3−^ (mg·L^−1^) in the medium: 443 (C) and 110 (P) Inflow (L·d^−1^): 10 (C) and 40 (P) Outflow (L·d^−1^): 10 (C) and 10 (P) Permeate (L·d^−1^): 30 (P)	Lab scale, perfusion mode (P, with a filtration step using a 800 kDa membrane) and continuous mode (C) Pretreatment: filtration (0.2 μm); UV–sterilisation; addition of NO_3_^−^ and PO_4_^3−^ Light (μE ·m^−2^·s^−1^): 525 Light/dark cycle: n.s Duration (d): 7 Temperature (°C): 25	Output: Biomass (g·L^−1^): 0.95 (C) and 0.53 (P) Dry weight productivity (g·L^−1^·d^−1^): 0.53 (C) and 0.29 (P)	Parameters for zero order removal kinetics: k_0 order_ NO_3_^−^ (mg·L^−1^·d^−1^): 480 (C) and 308 (P) k_0 order_ PO_4_^3−^ (mg·L^−1^·d^−1^): 60 (C) and 30 (P)	(Böpple et al. 2025)
*Chlorella* in mono (M) and co-culture (1:1) (C) with *Scenedesmus*	Effluent of slaughterhouse Initial composition: COD (mg·L^−1^): 905 – 919 NH_4_^+^ (mg·L^−1^): 330 – 365 NO_3_^−^ (mg·L^−1^): 89 – 102 TN (mg·L^−1^): 359 – 376 PO_4_^3−^ (mg·L^−1^): 61 – 88 TP (mg·L^−1^): 93 – 105	Bioreactor, batch mode Light (μE ·m^−2^·s^−1^): 150—300 Light/dark cycle (h/h): 12/12 Duration (d): 20 Temperature (°C): 28.2 − 32.5	Output: Biomass (g·L^−1^): 0.11 (M), 0.09 (C)	Parameters for zero-order removal kinetics: k_0 order_ COD (mg·L^−1^·d^−1^): 41.82 (M), 42.37 (C) k_0 order_ NH_4_^+^ (mg·L^−1^·d^−1^): 18.42 (M), 18.5 (C) k_0 order_ TN (mg·L^−1^·d^−1^): 20.8 (M), 20.95 (C) k_0 order_ PO_4_^3−^ (mg·L^−1^·d^−1^): 3.32 (M), 3.38 (C) k_0 order_ TP (mg·L^−1^·d^−1^): 4.67 (M), 4.625 (C)	(Bedane and Asfaw 2023)
*Chlorella vulgaris*	Effluent of aquaculture Initial composition: COD (mg·L^−1^): 2700 NH_4_^+^ (mg^·^L^−1^): 1.9 NO_2_^−^ (mg^·^L^−1^): 0.9 TN (mg^·^L^−1^): 84.43 PO_4_^3−^ (mg^·^L^−1^): 3.44 pH: 7.6	Lab scale, batch mode Light: n.s Light/dark cycle (h/h): 12/12 Duration (d): 21 Temperature: n.s		Parameters for first-order removal kinetics: k_1st order_ COD (d^−1^): 0.0823 k_1st order_ NO_3_^−^ (d^−1^): 0.0248 k_1st order_ NO_2_^−^ (d^−1^): 0.0629 k_1st order_ PO_4_^3−^ (d^−1^): 1.02	(Aranda-Vega et al. 2024)
*Chlorella* sp. and *Phaeodactylum tricornutum*	Effluent of grouper aquaculture (GAW) and fish and shrimp mixed aquaculture (FSAW) and synthetic aquaculture (SA) Initial composition: COD (mg·L^−1^): n.d. (SA), 13.76 (GAV) and 9.88 (FSAV) NH_4_^+^ (mg·L^−1^): 10 (SA), 5.65 (GAV) and 6.92 (FSAV) NO_2_^−^(mg·L^−1^): 2.50 (SA), 0.55 (GAV) and 5.25 (FSAV) NO_3_^−^ (mg·L^−1^): 2.50 (SA), 5.70 (GAV) and 3.13 (FSAV) TN (mg·L^−1^): 15.00 (SA), 13.82 (GAV) and 16.56 (FSAV) PO_4_^3−^ (mg·L^−1^): n.d. (SA), 1.60 (GAV) and 1.80 (FSAV) TP (mg·L^−1^): 1.25 (SA), 1.72 (GAV) and 1.93 (FSAV) pH: 8.0 (SA), 7.85 (GAV) and 7.86 (FSAV)	Lab scale (SA) and outdoor reactor (GAW and FSAW), batch mode Light (lx): 6000 (lab scale) and 9988 (average, pilot plant) Light: dark cycle (h/h): 12:12 (lab scale) and natural (pilot scale) Duration (d): 14 (lab scale), 5 (pilot plant) Temperature (°C): 25 (lab scale) and 31.1 (average, pilot plant)	Parameters for the simplified Lotka-Volterra model: (at the optimal ratio *Chlorella* sp.*: Phaeodactylum* = 1:8, in SA) i = *Chlorella* sp.*;* j = *P. tricornutum* *β*_*i*_: 10.32, *β*_*j*_: 0.05 K_1_ (cell·mL^−1^): 9.91·10^6^ K_2_ (cell·mL^−1^): 16.4·10^6^ Output: Biomass (g·L^−1^): not reported (SA), 0.22 (GAW) and 0.24 (FSAW) (pilot plant) Protein (%): 35.3 (SA), 33.52 (GAW) and 33.52 (FSAW) Lipid (%): < 5 (SA), 21.68 (GAW) and 24.19 (FSAW)	Removal efficiency: NH_4_^+^ (%): 100 (SA), 100 (GAV) and 96.71 (FSAW) TN (%): >90 (SA), 88.24 (GAV) and 88.48 (FSAW) TP (%): >90 (SA), 95.20 (GAV) and 97.12 (FSAW)	(Ding et al. 2024)
*Chlorella vulgaris* and *Scenedesmus acutus* co-culture	Swine wastewater Initial composition: COD (mg·L^−1^): 808.00 TN (mg·L^−1^): 808.08 TP (mg·L^−1^): 70.96 pH: 7.87	Lab scale, batch mode Pretreatment: anaerobic digestion Light (natural, μE ·m^−2^·s^−1^): 97 at 12:00 pm and 3 at 6:00 pm Light: dark cycle (h/h): 13/11 Duration (d): 30 Temperature (°C): 30	Parameters for modified Lotka-Volterra model: i = *C. vulgaris*; j = *S. acutus* *μ*_*i*_ (d^−1^): 0.81 β_ii_ (cell^−1^·mL·d^−1^): − 0.006; β_jj_ (cell^−1^·mL·d^−1^): 2.23; β_ij_ (cell^−1^·mL·d^−1^): 1.99; β_ji_ (cell^−1^·mL·d^−1^): − 0.07 Output: Biomass (g·L^−1^): 2.88 Lipid (%): 6.3	Removal kinetics COD (mg·L^−1^·d^−1^): 134 TN (mg·L^−1^·d^−1^): 93.3 TP (mg·L^−1^·d^−1^): 8.88 Removal efficiency: COD (%): 89 TN (%): 56 TP (%): 67	(Palafox-Sola et al. 2023)
*Chlorella vulgaris*, and bacteria (from dairy sludge) at the ratio 2:1	Effluent of dairy products Initial composition: COD (mg·L^−1^): 800 NO_3_^−^ (mg·L^−1^): 63 PO_4_^3−^ (mg·L^−1^): 17.24 pH: 7.8	Lab scale, batch mode Light (lx): 28,000–40000 Light/dark cycle: n.d Duration (d): 8 Temperature (°C): 28–30	Parameters for modified Lotka-Volterra model: i = *C. vulgaris*; j = bacteria *μ*_*i*_ (d^−1^): 2 β_ii_ (cell^−1^·mL·d^−1^): 0.5; β_jj_ (cell^−1^·mL·d^−1^): 0.5; β_ij_ (cell^−1^·mL·d^−1^): 0.7; β_ji_ (cell^−1^·mL·d^−1^): 0.7 Output: Biomass (g·L^−1^): 1.52	Removal efficiency: COD (%): 85 NO_3_^−^ (%): 75 PO_4_^3−^ (%): 80	(Roy et al. 2023)

COD: chemical oxygen demand; TN: total nitrogen; TP: total phosphorous. The equations and their relative physiologically relevant parameters are reported in Table 1

### Application of the logistic model

The logistic model provided a slightly better fit than the Gompertz equation in some studies (Kothari et al. 2024; Carbonell Sorí et al. 2025), while showing comparable performance in others (Širić et al. 2023). It has also been applied to optimize the treatment of complex substrates, such as nejayote and swine wastewater (López-Pacheco et al. 2019) (Table 2).

### Application of the Verhulst model

The Verhulst model, which does not account for the lag phase, has been applied only in a limited number of studies, specifically to investigate the treatment of cassava-processing effluent using *Chlorella sorokiniana* in either monoculture or in co-culture (Padri et al. 2022, 2025). Notably, the Verhulst model proved useful for transferring the kinetic parameter μ_max_ of 0.35 d^−1^, obtained from laboratory-scale batch cultures, to semi-continuous systems, resulting in θ_opt_ of 5 d, and thereby supporting process scale-up (Padri et al. 2025) (Table 2).

### Application of the exponential growth curve

When growth was assumed to follow a simple exponential trend, the values of μ for *Chlorella* spp. cultivated on food industry effluents on lab-scale typically fell in the range 0.115–0.18 d^−^^1^, depending on the substrate (cheese, tequila vinasse, shrimp wastewater, seafood wastewater) (Gao et al. 2018; Nasir et al. 2023; Najar-Almanzor et al. 2024) (Table 2). These values corresponded to θ_opt_ of 11–17 d. The complexity of the substrate plays a dominant role in growth parameters, since under optimized synthetic conditions, *C. sorokiniana* reached much higher μ (up to 0.61 d^−1^) (Gupta and Pawar 2019). Interestingly, industrial-scale cultures of *C. vulgaris* in corn molasses, which is a glucose-rich substrate, under fed-batch mode to stabilize ammonium concentration and pH, achieved a value of μ as high as 1.0 d^−1^ (Trovão et al. 2025). A high μ of 0.98 d^−1^ was also found in pilot plants with *Chlorella* spp. cultures performed in batch mode for the treatment of starch and alcohol (Tan et al. 2018) (Table 2). Outdoor cultivation of *C. pyrenoidosa* in soybean effluents in batch mode achieved even higher values for μ, but a large fluctuation was observed from 0.73 in spring to 1.59 d^−1^ in summer (Tan et al. 2021) (Table 2).

### Overview of *Chlorella* growth

Several studies summarized in Table 2 have highlighted the critical role of the lag phase, which is often caused by the limited bioavailability of the C source present in certain food-industry effluents. It is evident that this phase should be minimized through cultivation strategies or chemical and enzymatic pretreatment approaches.

**Fig. 1:**
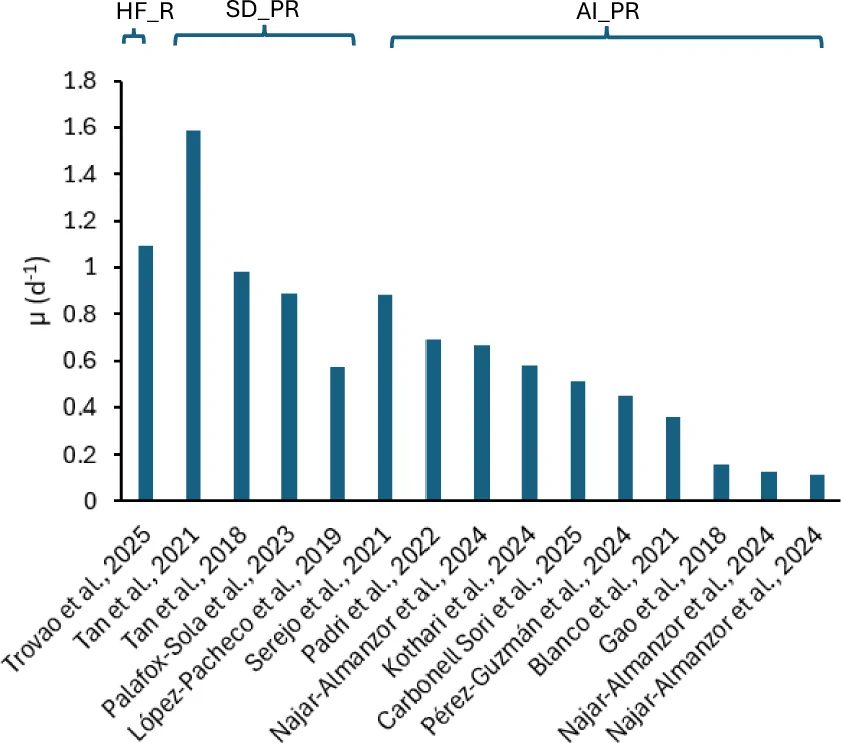
Growth rates of *Chlorella* spp. cultivated in food- and beverage industry effluents. HF_R = heterofermentation reactor; SD_PR = sun-driven photobioreactor; AI_PR = artificially illuminated photobioreactor

Assuming that this limitation can be effectively overcome, and considering growth rate as the sole performance indicator, reported values range from 0.115 to 1.59 d^−^^1^ (Fig. 1). These values provide a useful baseline for future studies aimed at improving process efficiency and cultivation performance.

## Kinetic models integrating with environmental factors

Kinetic models integrating environmental factors include those based on single limiting substrates, such as the Monod and Andrews models, the one tailored to light availability, such as the Huisman model, and the more comprehensive multifactorial approaches. The Monod model (Eq. 8, Table 1) has been applied to describe algal growth as a function of the concentration of a single limiting substrate, assuming that the variations in other nutrients have negligible effects. This model follows a saturation-type kinetic behavior, analogous to Michaelis–Menten enzyme kinetics, where μ increases hyperbolically with substrate concentration until reaching a maximum rate (Monod 1949). The substrate concentration at which the growth rate is half of its maximum is defined as the affinity constant (K_s_ in Eq. 8, Table 1). However, the Monod model does not account for potential inhibitory effects at high substrate concentrations. To address this limitation, the Andrews model (Eq. 9, Table 1) incorporates an inhibition constant (K_is_ in Eq. 9, Table 1), enabling the description of μ decline at elevated substrate levels and allowing determination of the optimal substrate concentration (S_opt_ in Eq. 10, Table 1) for maximal growth (Andrews 1968).

Light availability has been identified as a limiting factor in photobioreactors, especially as biomass concentration increases and the medium becomes more turbid. Variations in light paths caused by different reactor geometries can further influence overall productivity (Böpple et al. 2025). To capture these dynamics, the Huisman model (Eqs. 11—13, Table 1) was developed to describe how light governs algal growth in continuous systems (Huisman et al. 1999, 2006). This model identifies a critical light intensity (I_in_ in Eq. 12, Table 1) that determines whether algal populations grow or decline through its influence on a light-dependent parameter (a_H_ in Eq. 11 and 12, Table 1). When the incoming light intensity exceeds the critical light intensity, the algal population expands, but if it falls below it, the population decreases. Notably, *Chlorella* exhibits a particularly low critical light intensity of 2.75 μE·m^−2^·s^−1^, which is much lower than that of other algae, such as *Scenedesmus* that requires 6.49 μE·m^−2^·s^−1^ (Huisman et al. 1999). This distinction is important in competitive environments, as algal species with lower critical light requirements are predicted to outcompete others regardless of initial conditions (Huisman et al. 1999). This model also highlights the role of background turbidity (k_bg_ in Eq. 11, Table 1), i.e., light penetration diminishes when turbidity increases, leading to reduced algal growth. Despite the importance of the Huisman model, it has not been used in any of the *Chlorella* applications reported so far.

Multifactor models can capture the combined influence of multiple variables and their interaction effects. In this context, the second-order polynomial quadratic equation (Eq. 14, Table 1) has generally been applied since it provided a satisfactory fit with the experimental data. In this model, positive coefficients indicate factors that can enhance growth, whereas negative coefficients reflect inhibitory effects. The linear coefficients (α_n_ in Eq. 14, Table 1) describe the impact of a factor at low concentrations, while the quadratic coefficients (α_nn_ in Eq. 14, Table 1) represent their influence at higher concentrations. The interaction coefficient α_nm_ in Eq. 14 (Table 1) quantifies how two factors jointly affect the response, revealing whether their combined effect is synergistic or antagonistic.

### Application of Monod and Andrews models for the effects of N and P sources

The Monod model has been used to describe algal growth as a function of N or P availability. In food processing effluents, these nutrients are typically present at high concentrations and do not generally limit the initial growth of microalgae. However, as cultures approach the end of the exponential phase in batch reactors, N and P can become depleted, leading to marked changes in growth kinetics and biomass composition (Eriksen et al. 2007). In a lab-scale study on swine wastewater treatment using *C. vulgaris*, Monod constants of 2.27 mg·L^−^^1^ for COD, 2.28 mg·L^−^^1^ for TN, and 2.31 mg·L^−^^1^ for TP were obtained at pH 7.87 (Palafox-Sola et al. 2023) (Table 2). However, these results have not yet been validated at larger scales.

The Andrews kinetic model (Eq. 9, Table 1) was applied to determine the optimal NH_4_^+^ concentration of *C. vulgaris* cultivated on gelatin wastewater. This approach identified an optimum NH_4_^+^ concentration of 37.59 mg L^−1^ at pH 7.5 at the lab scale and later validated in a bubbled photobioreactor (Blanco et al. 2021) (Table 2). It is important to note, however, that this value is substrate-specific and cannot be generalized, as the optimal NH₄⁺ level depends strongly on pH, which governs the NH₄⁺/NH₃ equilibrium (Tam and Wong 1996). In fact, compared to the S_opt_ found at pH 7.5 (Blanco et al 2021), the S_opt_ was found to be six times higher at pH 7.0 (Tam and Wong 1996), where, due to the displacement of the chemical equilibrium of the reaction, all ammoniacal N was present in the ionic form of NH_4_^+^, which is less toxic to microalgae.

### Application of multifactor models for the effects of nutrients, light, and temperature

Microalgae cultivated in food and beverage industry effluents typically grow under mixotrophic conditions without the addition of external C sources (Table 2). However, hydroponic wastewater represents a notable exception: although it contains low organic load, it is rich in NO_3_^−^ and PO_4_^3−^. For this reason, two lab-scale studies evaluated the heterotrophic cultivation of *Chlorella* sp. in hydroponic lettuce effluent by testing different concentrations of glucose as the external C source and indole−3-acetic acid as a phytohormone, using a second-order polynomial quadratic model (Pekkoh et al. 2024) (Table 2). The model identified comparable optimal concentrations of glucose (10.89 and 12.2 mg·L^−^^1^, respectively) and indole−3-acetic acid (10.83 and 15 mg·L^−^^1^), which minimized osmotic stress or oxidative damage (Pekkoh et al. 2024).

The concentrations of TN and TP are present at high levels in food and beverage industrial effluents. Only one lab-scale study, conducted on poultry-slaughterhouse effluent, evaluated TN and TP as key variables for algal growth together with photoperiod duration and incubation time, identifying optimal levels of 425 and 65 mg·L^−1^, respectively (Dehghan Banadaki et al. 2024) (Table 2).

Light exposure is a relevant key factor as algal growth depends on the balance between light availability and nutrient supply that drives cultures toward either photoautotrophic-oriented or heterotrophic-oriented metabolism. Across studies on food processing wastewater treatment using *Chlorella* spp., light intensities typically ranged from 0 to 800 μE·m^−^^2^·s^−^^1^ (or 0–50,700 lx) (Table 2). In lab-scale approaches aimed at identifying maximum biomass growth, light intensity was combined with dairy wastewater concentration and temperature (Kumari et al. 2022) or with effluent marine aquaculture concentration and photoperiod (Zhao et al. 2024) or with pH, temperature, and agitation speed (Dhandayuthapani et al. 2022), but results were not scaled-up (Table 2).

A second-order polynomial model was used to evaluate the combined effects of light intensity (100–800 µE·m^−^^2^·s^−^^1^) and temperature (20–40 °C) on the lab-scale growth of *Chlorella* sp. cultivated in soy sauce and defective soy sauce wastewaters, with results validated at pilot scale (Kim et al., 2023; Table 2).

In general, light intensity emerged as the most influential growth factor. Adequate illumination enhances the growth rate and photosynthetic efficiency of microalgae. However, microalgal growth is also constrained by illumination above the saturation threshold, potentially leading to cell death (Patel et al. 2019; Bialevich et al. 2022).

### Overview of environmental factors affecting *Chlorella* growth

Studies reported in Table 2 indicate that the kinetic parameters derived from different models (e.g., Monod and Andrews) and the optimal conditions identified through multifactorial models are extremely important for optimization, but they are highly specific to the improvement of the treatment of a particular food effluent and cannot be readily transferred to different systems or operating conditions.

**Fig. 2:**
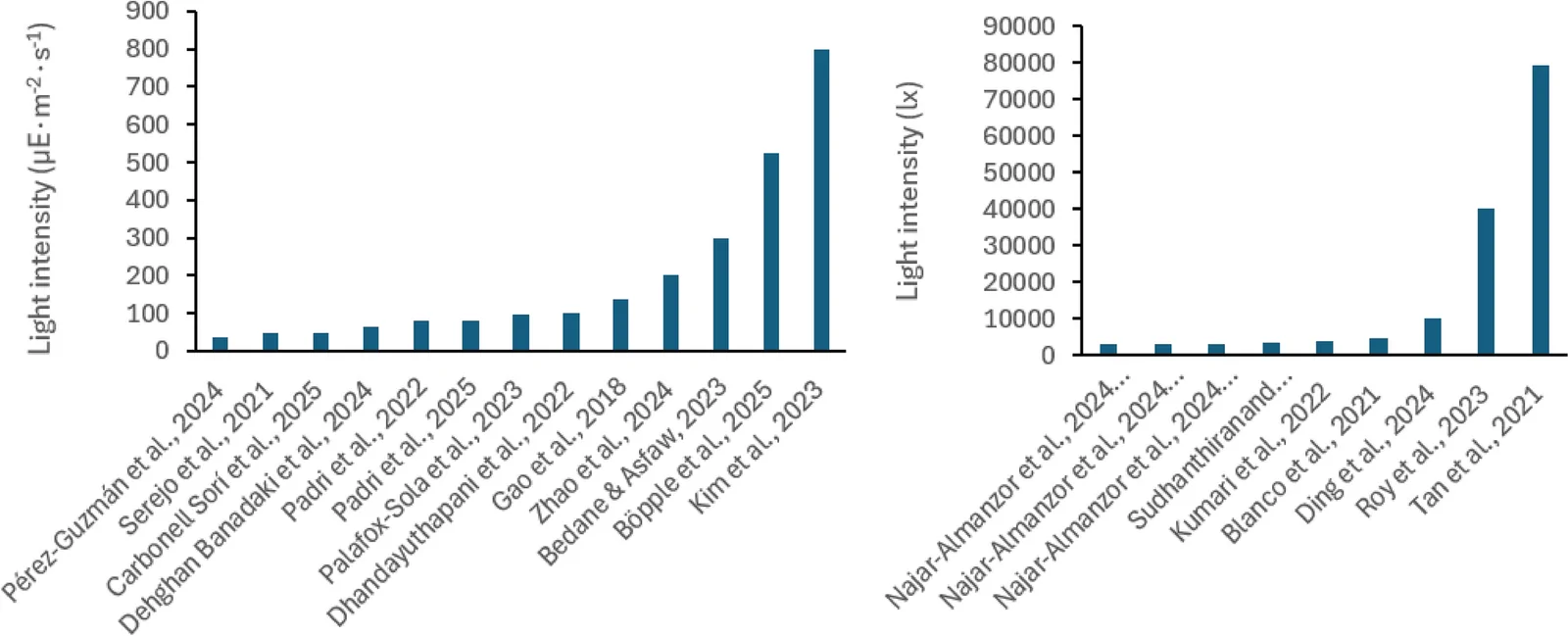
Light intensity applied in photobioreactors for the cultivation of *Chlorella* spp. in food- and beverage industry effluents

A key challenge that emerges is light availability, which has been reported differently across studies (Fig. 2). To enable the establishment of reliable reference values, light intensity should first be expressed by standardized units, both for natural sunlight and artificial illumination. This would facilitate meaningful comparisons among studies and improve the reproducibility of experimental results. Illuminance, expressed in lux, is weighted according to the sensitivity of the human eye, which is greatest in the green region of the visible spectrum. Therefore, illuminance cannot be directly converted into photosynthetic photon flux density (PPFD), expressed in µmol·m^−^^2^·s^−^^1^, without knowing the spectral distribution of the light (Blanken et al. 2013). While illuminance is appropriate for assessing human visual perception and visual comfort, PPFD is recommended for studies of algae and photosynthetic processes. Furthermore, the Huisman model has not been considered in the studies reviewed, despite its potential value for guiding the scale-up of microalgal cultivation systems and supporting the development of large-scale applications.

## Kinetic models for algal products’ accumulation

Algal biomass composition can vary significantly depending on the substrate and cultivation conditions. For example, when the culture medium contains N in readily assimilable forms, such as nitrates, nitrites, urea, or ammonium, microalgae tend to channel metabolism toward protein synthesis. In contrast, N‐depleted conditions redirect metabolic fluxes toward storage compounds, such as starch or lipids (Eriksen et al. 2007; Yaakob et al. 2021; Maltsev et al. 2023). Importantly, the accumulation of specific high-value compounds does not necessarily correlate with cell growth.

Lipids are among the most valuable products obtained from algal cultivation, particularly due to their potential use as biofuels, with a high proportion of saturated fatty acids generally improving biodiesel performance (Tan et al. 2021). Lipid contents, ranging from 6.3 to 30.7%, have been reported for *Chlorella* spp. cultivated at lab scale in food and beverage industry wastewaters (Table 2). High lipid concentrations ranging from 25.4 to 28.7% were observed in *Chlorella* sp. cultivated on a lab scale in seafood processing wastewater, which also exhibited a fatty acid profile well suited for biodiesel production (Gao et al. 2018). A high lipid level, equal to 30.7%, was also observed in *C. vulgaris* biomass cultivated on slaughterhouse wastewater on a lab scale, likely because of N stress caused by the low nitrate concentration (149 mg·L^−1^). However, the lipid profile was not reported (Kothari et al. 2024). A low N level was later confirmed to be associated with increased lipid content in *Chlorella* biomass (Garza-Leon et al., 2026). The outdoor co-culture of *Chlorella* sp. and *P. tricornutum* grown on effluents from grouper aquaculture and mixed fish-shrimp aquaculture yielded biomass containing 21–24% lipids, which was substantially higher than lab-scale values, likely due to stress-induced lipid accumulation. Furthermore, the fatty acid profile of this biomass showed strong potential for biodiesel production (Ding et al. 2024).

Carbohydrates in algal biomass are particularly attractive as substrates for bioethanol production because they lack lignin and their cells are not recalcitrant, reducing the need and cost of pre-treatment. Notably, *C. vulgaris* cultivated in wastewater from industrial oil processing achieved a carbohydrate content of 40.19% (Silva et al. 2022). However, in most studies conducted on wastewater from the food and beverage industry, the carbohydrate content of the resulting biomass was not disclosed (Table 2). Reported values include 18–20% in *Chlorella* grown in soybean-processing effluents using a reactor for outdoor cultivation in batch mode (Tan et al. 2021).

The protein fraction of *C. vulgaris* features a well-balanced amino acid profile and exhibits functional properties, including emulsifying, water-holding, and gel-forming abilities, that make it attractive for diverse food applications (García-Encinas et al. 2025). Notably, *C. pyrenoidosa* cultivated in soybean-processing effluent achieved a protein content of 60% in a reactor for outdoor cultivation in batch mode (Tan et al. 2021).

Algal pigments, such as chlorophylls, carotenoids, and phycobiliproteins, have received attention as eco-friendly colorants for the food industry (Sun et al. 2023; Ma et al 2021). These pigments are typically produced when microalgae grow under autotrophic conditions (Patel et al. 2019). Consequently, on a lab scale, *C. vulgaris* produced 5.90 mg⋅g^−1^ of pigments when cultivated autotrophically in the traditional BBM, while it produced 1.62, 0.36, and 0.22 mg⋅g^−1^ when grown mixotrophcally in nejayote, tequila vinasse, and cheese whey, respectively (Najar-Almanzor et al. 2024).

Limited information is available on the phenolic content of *Chlorella* spp. cultivated in food and beverage industry effluents. *Chlorella* spp. biomass cultivated in nejayote exhibited a total phenolic content of 2.87 mg⋅g^−^^1^ gallic acid equivalents (GAE), attributed to the presence of hydroxycinnamic, *p-*coumaric, ferulic, dehydrodiferulic, and dehydrotriferulic acids originally found in the corn-kernel-derived effluent (Najar-Almanzor et al. 2024).

However, the extent to which microalgae can actively uptake external phenolics and how much of the observed reduction results instead from abiotic degradation remains unclear.

This gap highlights the need for more comprehensive investigations into phenolic dynamics during algal cultivation (Andriopoulos and Kornaros 2024). To properly assess the role of algae in preserving or decreasing phenolic compounds as value-added products, it is essential to distinguish between the different pathways responsible for changes in their concentration. A comparative kinetic analysis of phenolic compound concentrations in the culture medium, both in the presence and absence of algae, would help differentiate algae-mediated transformations from abiotic degradation. In general, the abiotic degradation of phenolic compounds follows first-order kinetics, and rate constants reported in previous studies could provide useful reference values (Jorge et al. 2023).

In most studies, when *Chlorella* spp. is cultivated in food and beverage industry effluents, the production of value-added compounds was not kinetic modelled. In some cases, multifactorial polynomial quadratic models (Eq. 15, Table 1), which are also used for modelling cell growth, were employed to identify how different growth factors influence the yield of specific compounds. An enhanced Gompertz equation (Eq. 16, Table 1) has also been proposed to describe product formation; however, this method is applicable only when the desired output is produced concurrently with biomass growth.

### Application of the multifactorial model to optimize lipid production

In a lab-scale study on *C. vulgaris* cultivation, a polynomial quadratic model was used to examine how the concentrations of glucose (as the C source) and indole−3-acetic acid (as a phytohormone) added to hydroponic effluent influence lipid content, polyunsaturated fatty acids, and overall algal biomass. As expected, the conditions that maximized lipid content differed from those that maximized polyunsaturated fatty acids or biomass (Sriket et al. 2024). Therefore, it is essential to define a target output in advance so that cultivation conditions for *C. vulgaris* can be optimized to prioritize the desired product.

### Application of the enhanced Gompertz equation to optimize biohydrogen productivity

In another lab-scale study, *C. vulgaris* cultivated on palm-kernel expeller waste was used to produce biohydrogen through dark fermentation. A major limitation of this application lies in the substrate’s lignin-rich matrix, where lignin is tightly associated with cellulose and hemicellulose, reducing its biodegradability and hindering microbial depolymerization. For this reason, a thermal pretreatment step through heating the substrate in water at a controlled temperature and duration is required to solubilize lignin and hemicellulose and improve subsequent fermentability. The overall process integrated both the pretreatment and the dark-fermentation stages and was modelled using an enhanced form of the modified Gompertz equation, in which pretreatment duration (l in Eq. 16, Table 1) was introduced as an additional variable. In this adaptation of the model, hydrogen production (H in Eq. 16) replaced cell concentration (Y in Eq. 1), making H a function of both fermentation time and pretreatment duration. Similarly, the growth-related parameter μ_max_ was substituted with the maximum hydrogen production potential (P_max_) and the maximum hydrogen production rate (R_max_). Model predictions identified a pretreatment duration of 1 h as the optimal condition. Experimentally, this corresponded to a hydrogen yield of 387.9 mg⋅g^−^^1^ when the substrate was thermally pretreated at 90 °C for 1 h (Ahmad Sobri et al. 2023).

### Overview of algal products’ accumulation

The accumulation of high-value-added products by *Chlorella* grown on food and beverage industry effluents remains largely unexplored from a kinetic perspective. In addition to yield, potential issues related to contamination and downstream purification processes also remain to be defined.

## Kinetic models for pollutant removal

Several studies have shown that *Chlorella* spp. monocultures possess strong phycoremediation capabilities in various food and beverage industry wastewaters that were not sterilized before treatment. In these systems, indigenous bacteria present in the raw effluents likely contributed to the observed removal efficiencies for COD and inorganic N and P (Palafox-Sola et al. 2023). As a result, the rate at which contaminants are removed does not always correspond directly to the algal growth rate. Nonetheless, studies consistently reported removal efficiencies above 80% for COD, TN, and TP, confirming that *Chlorella* is highly adaptable to a wide variety of wastewaters (Table 2).

The time-course of pollutant removal by algal cultures has been examined in only a limited number of studies. In one approach, the Monod model, which is traditionally used to relate microbial growth to a single limiting nutrient, was reformulated by replacing the specific growth rate with the contaminant removal rate (Eq. 17, Table 1). Other studies reported that pollutant concentrations decreased according to either zero-order or first-order kinetics (Eqs. 18 and 19, Table 1). Additionally, the polynomial multifactor model previously used to relate operational variables to algal growth or product formation has also been extended to describe how these factors influence phycoremediation performance (Eq. 20, Table 1).

### Application of the Monod model for pollutant removal

In one study, the Monod model was applied to describe the removal kinetics of contaminants from dairy effluent treated with *C. pyrenoidosa* under lab-scale conditions. The affinity constants for NO₃⁻, NH₄⁺, and PO₄^3^⁻ were determined to be 16.21, 19.12, and 5.41 mg·L^−1^, respectively, at pH 7.0 (Das et al. 2022). In contrast, another study investigating *C. vulgaris* grown on dairy wastewater reported much higher affinity constants—198.77 mg·L^−1^ for TN and 668.09 mg·L^−1^ for TP at pH 7.97 (C. and Perumalsamy 2024). These large differences suggest that the model cannot reliably capture general trends, likely because culture conditions, such as pH, strongly influence the dissociation equilibria of inorganic N and P species.

### Application of zero-order and first-order kinetic models for pollutant removal

A few lab-scale studies have applied the zero-order or the first-order kinetic models to analyze the bioremediation performance of *Chlorella* spp. In batch cultures, zero-order removal rates for COD were 10 mg⋅L^−^^1^⋅d^−^^1^ in cassava-processing wastewater (Padri et al. 2025), 41.82 mg L^−^^1^⋅d^−^^1^ in slaughterhouse effluent (Bedane and Asfaw 2023), and 89.9 mg·L^−^^1^⋅d^−^^1^ in anaerobically digested swine wastewater (Palafox-Sola et al. 2023). In the same batch mode studies, zero-order removal rates for TN and TP were 55.0 and 9.72 mg·L^−^^1^·d^−^^1^ (Palafox-Sola et al. 2023), 1.0 and 1.1 mg·L^−^^1^·d^−^^1^ (Padri et al. 2025), and 20.8 and 4.67 mg·L^−^^1^·d^−^^1^ (Bedane and Asfaw 2023). Overall, these studies indicated that removal rates generally increased slightly when co-cultures were employed.

Increased phycoremediation efficiency was obtained by continuous or perfusion mode cultures of *C. vulgaris* in salmon aquaculture effluent, which achieved NO_3_^−^ removal rates of 480 and 380 mg·L^−^^1^·d^−^^1^, respectively, and PO_4_^3−^ removal rates of 60 and 30 mg·L^−1^·d^−1^, respectively (Böpple et al. 2025) (Table 2).

First-order rate constants for COD removal were 0.0429 d^−1^in slaughterhouse effluents (Kothari et al. 2024) and 0.0823 d^−^^1^ in the effluent of aquaculture (Aranda-Vega et al. 2024). For NO_3_^−^ and PO_3_^4−^, first-order removal rates were 0.0508 and 0.0450 d^−1^ in slaughterhouse effluents (Kothari et al. 2024) and 0.0248 and 1.02 d^−1^ in the effluent of aquaculture (Aranda-Vega et al. 2024). Higher removal rates—approximately one order of magnitude greater were achieved using a co-culture of *C. reinhardtii* and *Fischerella muscicola* to treat shrimp-processing effluent (Bhatt et al. 2024).

### Application of multifactor kinetic models for pollutant removal

To optimize the treatment of dairy-industry effluent using *C. vulgaris*, three operational factors, including wastewater concentration (0–100%), temperature (20–30 °C), and light intensity (2000–4000 lx), were systematically adjusted. Under the optimal conditions, the process achieved high phycoremediation performance, removing 96.65% of BOD and 87.50% of COD (Kumari et al. 2022) (Table 2). Moreover, a positive interaction was observed between temperature and light intensity, as well as between wastewater concentration and light intensity (Kumari et al. 2022). The removal of TN and TP from marine aquaculture wastewater using *Chlorella* sp. cultivated in an integrated photobioreactor system was evaluated under varying light intensities, photoperiods, and inoculum concentrations. All three factors significantly influenced TN and TP removal rates. Regarding interaction effects, a reciprocal interaction between inoculum concentration and light intensity was observed for TN removal, whereas the interaction between inoculum concentration and photoperiod was significant for TP removal (Zhao et al. 2024).

### Overview of phytoremediation activity

**Fig. 3:**
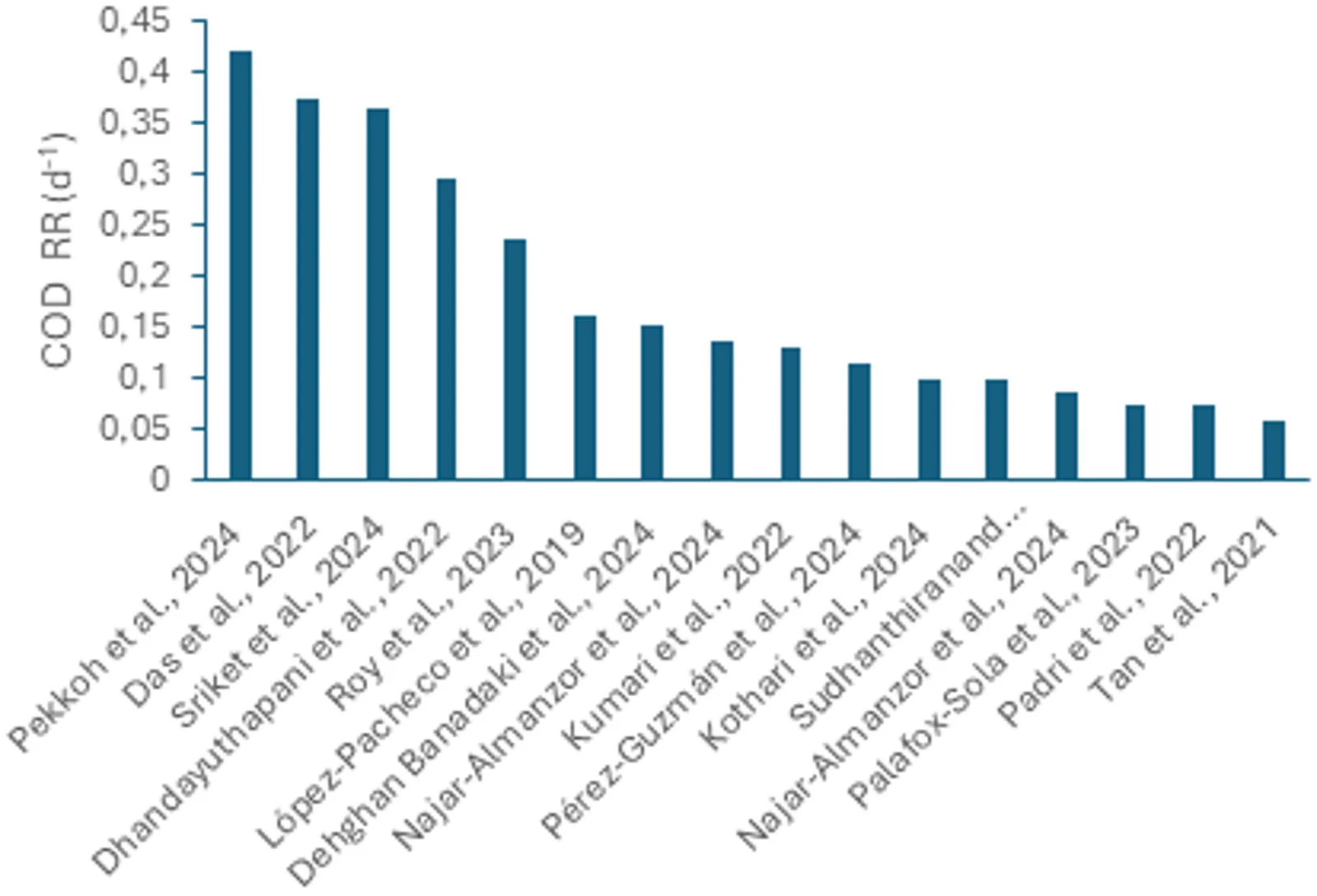
COD removal rate by *Chlorella* spp. cultivated in food and beverage industry effluents assuming first-order kinetics.

**Fig. 4:**
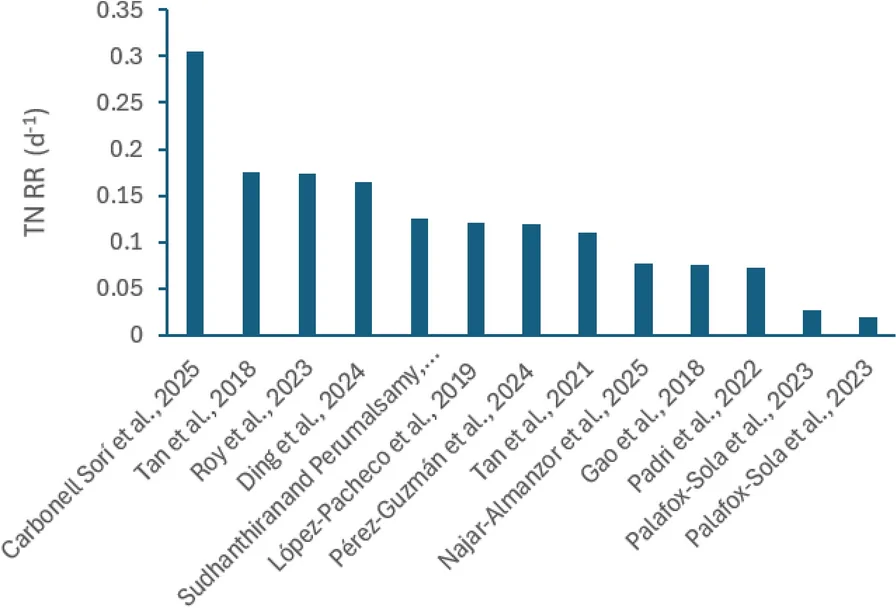
TN removal rate by *Chlorella* spp. cultivated in food and beverage industry effluents assuming first-order kinetics

**Fig. 5:**
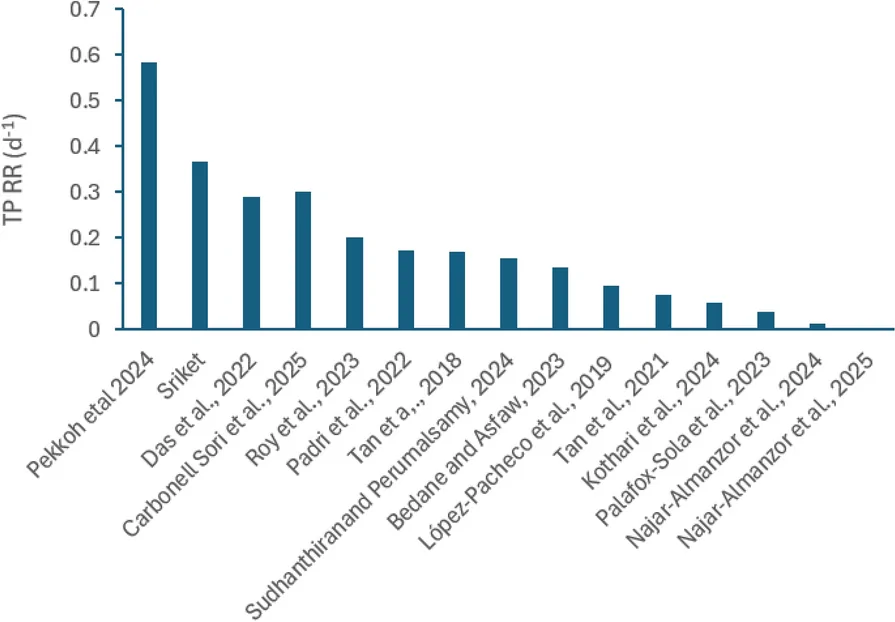
TP removal rate by *Chlorella* spp. cultivated in food and beverage industry effluents assuming first-order kinetics

In an industrial setting, real-time monitoring of contaminant concentrations is essential to regulate influent and effluent flow rates and ensure that discharge limits are consistently met. To support this, data on contaminant removal rates are necessary. Figures 3, 4 and 5 summarize the average removal efficiencies of COD, TN, and TP achieved by *Chlorella* spp. grown in food and beverage industry effluents, calculated assuming a first-order kinetics. The reported values represent a baseline for identifying potential improvement targets.

## Kinetic models of microbial interactions in co-cultures

The use of synergistic microbial communities for treating effluents from the food and beverage industry can enhance algal biomass production and improve pollutant removal compared with monocultures. In addition to employing preliminary steps, such as anaerobic digestion by bacteria, co-cultivating multiple microorganisms within a single process can further maximize overall efficiency. *C. sorokiniana* growth in cassava-processing effluent was enhanced by the presence of *S. thermocarboxydus*. Moreover, analyzing the full microbial growth profile revealed a non-linear relationship between algal and bacterial populations. The bacteria rely on algal exudates for their development, while the microalgae begin producing these metabolites only in the late exponential phase. Consequently, a syntrophic association emerges once the algal culture reaches the stationary phase, enabling mutually beneficial growth dynamics (Padri et al. 2025). These findings are particularly relevant for implementing co-cultures on an industrial scale. However, the synergistic interactions within the consortium have not yet been modelled.

Understanding how microbial species interact with one another and with their surrounding environment is crucial for optimizing algal growth. To support this, the classic Lotka–Volterra theory of predator–prey dynamics has been adapted to a wide range of ecological and biotechnological applications (Berryman 1992; Joseph et al. 2020). The simplest form of the Lotka-Volterra equation for two species, “i” and “j”, links the growth rate of species *i* to its intrinsic specific growth rate (μᵢ in Eq. 21, Table 1), the interaction exerted by species *j* (βⱼ in Eq. 21), and the carrying capacity of species *i* in the environment (K₁ in Eq. 21). Likewise, the growth rate of species *j* depends on its specific growth rate (μⱼ in Eq. 21), the interaction imposed by species *i* (βᵢ in Eq. 21), and its carrying capacity (K₂ in Eq. 21). However, this formulation incorrectly implies that each species would grow without bound in the absence of the other, an unrealistic assumption that highlights the need for model refinement (Berryman 1992). The classical Lotka-Volterra equation was later expanded with a self-limitation term to account for intraspecific interactions, i.e., the effects that individuals of the same species exert on one another. In this modified formulation, the growth rate of microorganism *i* and that of microorganism *j* depend not only on their specific growth rates (μᵢ and μⱼ in Eq. 22, Table 1, respectively), but also on interspecific interaction coefficients (βᵢⱼ and βⱼᵢ in Eq. 22, respectively) and intraspecific interaction coefficients (βᵢᵢ and βⱼⱼ in Eq. 22, respectively) (Berryman 1992). Positive interspecific coefficients indicate that the presence of species *j* enhances the growth of species *i*, whereas negative coefficients signify inhibition, which is an effect that becomes more pronounced at higher concentrations of species *j*.

### Application of the Lotka–Volterra model

The simplified Lotka–Volterra model was applied to describe the growth dynamics of two microalgae, *Phaeodactylum tricornutum* and *Chlorella* sp., during lab-scale treatment of marine-aquaculture effluent. The results showed that the competitive interaction between the two species strongly depended on their initial inoculum ratio. When a *Chlorella* sp. was inoculated at higher proportions (8:1 and 4:1 relative to *P. tricornutum*), the competition coefficients βⱼ were lower than K₁/K₂, whereas βᵢ exceeded K₂/K₁, indicating that *Chlorella* sp. inhibited the growth of *P. tricornutum*. Conversely, at lower *Chlorella* proportions (ratios between 2:1 and 1:8), βⱼ became greater than K₁/K₂ while βᵢ fell below K₂/K₁, suggesting that *P. tricornutum* inhibited the growth of *Chlorella* sp. (Ding et al. 2024). These interactions among the algae arise from the release of soluble algal products, such as polysaccharides, proteins, amino acids, and lipids, into the surrounding environment. These compounds regulate microalgal growth and shape the dynamics between species. In this system, the consortium achieved its highest bioremediation performance at an optimal ratio of 1:8 (Ding et al. 2024).

The modified Lotka-Volterra model was applied to analyze the interactions between *C. vulgaris* and *S. acutus* in a lab-scale co-culture grown on swine wastewater. The intraspecific parameter indicated that *C. vulgaris* population members exhibited a neutral interaction (βᵢᵢ = 0.006). In contrast, the interspecific coefficients showed that *S. acutus* positively influenced the growth of *C. vulgaris* (βᵢⱼ = 1.99), while *C. vulgaris* had little to no effect on *S. acutus* (βⱼᵢ = 0.007) (Palafox-Sola et al. 2023) (Table 2). In another study, the modified Lotka-Volterra model was used to examine the interactions between *C. vulgaris* and bacteria isolated from activated dairy sludge, cultivated together at a 2:1 ratio to treat cheese-processing effluent on a lab scale. The model revealed a mutually beneficial relationship between the two microorganisms (βᵢⱼ = βⱼᵢ = 0.7), attributed to the exchange of key metabolites such as CO₂ and O₂, which supported the growth and activity of both partners (Roy et al. 2023) (Table 2).

## Projected productivity and potential for large-scale contaminant removal

Most of the studies reported were conducted at the laboratory scale, and their practical implementation requires the design of efficient photobioreactors. Scale-up barriers should be overcome by investigating the effects of photobioreactor hydrodynamics on mass transfer, light availability, cell viability, and, ultimately, productivity. This is particularly critical for algal cultures because, as the cells proliferate, they increase the viscosity and opacity of the medium. Consequently, culture growth itself triggers mechanisms that hinder further growth. Moreover, the effectiveness of mechanical mixing, aeration, and pumping must be balanced against the algae’s tolerance to shear stress. A previous study showed that *C. vulgaris* exhibited higher specific growth and photosynthetic activity in a stirred-tank photobioreactor when subjected to agitation velocities of up to 1.26 m⋅s^−^^1^ (shear stress τ = 0.45 Pa), compared with cultures grown without agitation. However, further increases in mixing speed were detrimental because they caused cell lysis (Leupold et al. 2013). In light of these considerations, computational fluid dynamics (CFD) represents a valuable tool, as it can simulate complex flow patterns and turbulence within bioreactors, map light distribution, track the dispersed phase (i.e., bubbles) and the trajectories of simulated cells, and derive shear-stress profiles. This information can guide bioreactor design toward maximizing productivity while minimizing energy consumption, although the resulting design must, of course, be experimentally validated (Amanna et al. 2024). Assuming that the scale-up barriers have been overcome through CFD modelling and experimental validation, the productivity and contaminant-removal performance of the previously discussed algal treatments for food and beverage effluents (Table 2) were projected to the large scale. Several scenarios were developed using large-scale cultivation facilities as benchmarks (Tables 3 and 4). The first scenario focuses on heterotrophic algal biomass production. Under heterotrophic conditions, microalgae typically achieve high growth rates and generate biomass with elevated lipid content, though protein and pigment levels remain comparatively low. The main limitation of this approach lies in the indirect energy cost of the C sources (Barros et al. 2019). Using industrial effluents as nutrient sources would therefore be highly advantageous. At present, heterotrophic algal production is carried out at a Corbion facility in Brazil, co-located with a sugar mill that supplies the C source. However, since this plant cultivates microalgae belonging to the genera *Schizochytrium*, *Ulkenia*, and *Crypthecodinium* (Davis et al. 2021), it was not considered suitable as a benchmark for the present study. In one study of *C. vulgaris*, a two-stage process was developed in which biomass produced heterotrophically in industrial fermenters (5 m^3^, first stage) was directly used to inoculate an outdoor industrial-scale autotrophic production unit (second stage). The first stage achieved a yield of 31.86 g·L^−1^·d^−1^, corresponding to an annual productivity of 58 ton·y^−1^ (Barros et al. 2019), and was therefore considered a benchmark. Notably, heterotrophic cultivation of *C. vulgaris* on corn molasses reached a productivity of 22 g·L^−1^·d^−1^, which is close to this benchmark (Trovão et al. 2025). In contrast, the effluent from hydroponic lettuce cultivation, although suitable as a growth substrate, resulted in substantially lower heterotrophic yields (Pekkoh et al. 2024; Sriket et al. 2024).

**Table 3:** Projected efficacy indexes for *Chlorella* spp. cultivation in the effluents of food and beverage industry

Projected average annual productivity (P)	
$$P \left(ton\cdot {y}^{-1}\right)=\frac{B \left(mg\cdot {L}^{-1}\right)\cdot V \left(L\right)\cdot {10}^{-9}ton\cdot {mg}^{-1}\cdot 365 \left(d\cdot {y}^{-1}\right)}{t \left(d\right)}$$	
B (mg·L−1), biomass concentration at the end of the incubation;	
t (d) duration of the incubation;	
V (L), volume of the benchmark photobioreactor;	
365, working days per year	
Average removal rates for contaminants (RRc)	
$$R{R}_{c}\left({mg}_{c}{\cdot L}^{-1}\cdot {d}^{-1}\right)=\frac{ \left(0.01\cdot {RE}_{c}\right)\cdot {C}_{i} \left({mg}_{c}\cdot {L}^{-1}\right)}{t \left(d\right)}$$	
C, COD or TN or TP;	
REc (%), removal efficiency for c;	
Ci (mg·L), initial concentration of c in the effluent;	
t (d), duration of the incubation

**Table 4:** Comparison between benchmark plants and lab-scale/pilot-scale models based on projected productivity of algal biomass (P) and removal rates (RR) for contaminants

Benchmark 1: Heterotrophic fermenter (volume 5000 L) to produce a *Chlorella vulgaris* inoculum for an outdoor industrial autotrophic production unit of algal biomass rich in protein and pigments installed at All microalgae, Lisboa, Portugal. P (ton·y^−1^): 58. (Barros et al. 2019)		
Lab-scale model	P (ton·y^−1^)	Ref
*Chlorella vulgaris* in the effluent of hydroponic lettuce cultivation	40	(Trovão et al. 2025)
Benchmark 2: Tubular sun-driven photobioreactor (volume 448 m^3^·ha^−1^) for the mixotrophic production of *Chlorella vulgaris’s* biomass installed in Eindhoven, the Netherlands. P (ton·y^−1^·ha^−1^): 41. (Norsker et al. 2011)		
Lab-scale models	P (ton·y^−1^·ha^−1^)	Ref
*Chlorella pyrenoidosa* in the effluent of soybean processing	44—83	(Tan et al. 2021)
*Chlorella pyrenoidosa* in the effluent of starch and alcohol processing	28	(Tan et al. 2018)
*Chlorella pyrenoidosa* in the effluent of dairy products’ processing	17	(Das et al. 2022)
Benchmark 3: Tubular, artificially illuminated photobioreactor (volume 40.4 m^3^, occupying an area of 800 m^2^, irradiated surface 2161 m^2^) installed in Caltagirone, Sicily, Italy, to produce *Chlorella vulgaris*’s valuable products. P (ton·y^−1^): 1.2; E_L_ (kWh·kg^−1^): 40 (Gurreri et al. 2024)		
Lab-scale models	P (ton·y^−1^)	Ref
*Chlorella vulgaris* in the effluent gelatin processing supplemented with BBM; Light (lx): 4600; Light/dark cycle (h/h): 24/0	3.8	(Blanco et al. 2021)
*Chlorella sorokiniana* and natural communities of algae in the effluent of cassava processing; Light (μE ·m^−2^·s^−1^): 81; Light/dark cycle (h/h): 24/0	3.1	(Padri et al. 2022)
*Chlorella sorokiniana* and *Streptomyces thermocarboxydus* co-culture in the effluent of cassava processing; Light (μE ·m^−2^·s^−1^): 81 Light/dark cycle (h/h): 12/12	2.1	(Padri et al. 2025)
Benchmark 4: Tubular, artificially irradiated photobioreactor (volume: 400 L; light flow (I) 89 µE·m^−2^·s^−1^) to produce an inoculum of microalgae and bacteria for a large open raceway pond for effluent treatment, located at Inagro, Roeselare, Belgium.). RR (mg· L^−1^·d^−1^): 38 (COD); 4.2 (TN); 0.67 (TP). (Van Den Hende et al. 2014)		
Lab-scale models	RR (mg· L^−1^·d^−1^)	Ref
*Chlorella pyrenoidosa* in the effluent of starch and alcohol processing; natural light	COD: 299; TN: 28; TP: 3.2	(Tan et al. 2018)
*Chlorella pyrenoidosa* in the effluent of starch and alcohol processing; natural light (fall and spring)	COD: 60; TN: 20; TP: 2	(Tan et al. 2021)
*Chlorella pyrenoidosa* in the effluent of dairy products’ processing; natural light	COD: 86; TN: 9.2; TP: 2	(Das et al. 2022)
*Chlorella vulgaris*, and bacteria in the effluent of dairy products’ processing; light (lx): 28,000–40000	COD: 85; TN: 5.9; TP: 1.7	(Roy et al. 2023)
*Chlorella vulgaris* in the effluent of salmon aquaculture; Light (μE ·m^−2^·s^−1^): 525; Light/dark cycle: n.s	TN: 480; TP: 60	(Böpple et al. 2025)

The calculations of P, E_L_ and RR are reported in Table 3

All the other approaches proposed for treating food and beverage industry effluents with *Chlorella* spp. (Table 2) were carried out under mixotrophic conditions, using either sun-driven or artificially illuminated photobioreactors. Consequently, a second scenario was developed to evaluate the production of *Chlorella* spp. in sunlight-driven photobioreactors. The performance of sun-driven systems is expected to be strongly influenced by plant location, due to variations in global radiation, day length, and temperature. For this scenario, the benchmark system was a tubular, sunlight-driven photobioreactor (448 m^3^·ha^−1^) located in Eindhoven, the Netherlands, designed for algal biomass production and achieving yields of 41 tons·y^−1^·ha^−1^ (Norsker et al. 2011). It was assumed that the production of *Chlorella* spp. on food and beverage processing effluents could be scaled up to match the benchmark volume (448 m^3^), while neglecting location-specific environmental differences. Under this assumption, treating soybean-processing effluent with *C. pyrenoidosa* could exceed the target biomass yield (Table 4), also providing a notable protein content (approximately 55%) (Tan et al. 2021). Scaling up algal production on starch and alcohol processing effluents (Tan et al. 2018), swine-processing effluents (Palafox-Sola et al. 2023), and maize, nejayote, and swine effluents (López-Pacheco et al. 2019) would result in biomass yields slightly below the target (Tables 3 and 4), whereas all other evaluated approaches remained well below the benchmark performance.

Most studies on the treatment of food and beverage industry effluents using *Chlorella* spp. (Table 2) have been conducted under mixotrophic conditions in artificially illuminated photobioreactors. Building on this, a third scenario was developed to explore the potential scale-up of *Chlorella* spp. production in such systems. Artificial illumination enables tight control over algal growth, but it also requires a significant energy input. As a reference, the benchmark photobioreactor located in Caltagirone, Sicily (40.4 m^3^) is designed for the production of *C. vulgaris* with a productivity of 1.2 ton·y^−1^, which is intended for high-value applications, including biostimulants, lutein, and alternative proteins, and operates with an energy demand of 40 kWh per kilogram of biomass produced (Gurreri et al. 2024). Notably, some of the strategies proposed for treating food and beverage-industry effluents could, under the assumption of proportional efficiency, achieve benchmark productivity when scaled up to this reactor size. To achieve this result, process optimization followed two strategies. In one case, a synthetic medium was integrated into the effluent of gelatin processing (Blanco et al. 2021). In another case, co-cultures were applied (Padri et al. 2022; Padri et al. 2025).

Regarding the phycoremediation effect, no large-scale plants are currently in operation, likely due to the high cost of this technology (Acién et al. 2016). As a reference for large-scale performance, an artificially illuminated photobioreactor (400 L, 89 µE·m^−^^2^·s^−1^) designed to produce inoculum for an open raceway pond at Inagro (Roeselare, Belgium) was used as a benchmark. This system achieves removal rates of 38, 4.2, and 0.67 mg·L^−^^1^·d^−1^ for COD, TN, and TP, respectively (Van Den Hende et al. 2014). Interestingly, removal rates exceeding the benchmark values were observed for *C. pyrenoidosa* grown on the effluent of starch and alcohol processing (Tan et al. 2018), soybean processing (Tan et al. 2021) and dairy products’ processing (Das et al. 2022), as well as *C. vulgaris* and bacteria grown on the effluent of dairy products (Roy et al. 2023) and on the effluent of salmon aquaculture (Böpple et al. 2025) (Table 4).

## AI and ML approaches to describe kinetic processes in algal growth

Although multiple kinetic models have applied in kinetic modelling of *Chlorella* spp. for pollution control and resource recovery, traditional kinetic models are often inadequate to characterize non-linear, multi-variable environmental shifts, such as fluctuating light spectra, dynamic pH, and limiting nutrient states. Accounting for every biological interaction mechanistically could lead to over-parameterization. AI and ML are powerful tools to estimate hard-to-measure kinetic parameters in traditional models and can be used as powerful computational tools to overcome these limitations. AI and ML models can help identify complex relationships among cultivation parameters and avoid the limitations of traditional kinetic models. AI and ML have been used to improve various aspects of microalgae processes (Imamoglu 2024). A recent study developed a novel ML approach to model complex nonlinear relationships between cultivation conditions and lipid production in *Chlorella* species and predicted high algal lipid productivity (> 0.018 g·L^−1^·d^−1^) with an accuracy of 95.2% and offered a novel solution to reduce in the broad operational ranges of algal cultivation conditions significantly (by <96.7%) for improved lipid productivity (Lopez et al. 2026). This study also identified exogenous iron and P as key drivers of high lipid productivity, challenging the longstanding focus on N starvation as the dominant lipid trigger strategy in the literature, suggesting that AI and ML offer a promising avenue to boost algae lipid production through efficient, data-driven algal cultivation (Lopez et al. 2026).

The commonly used ML algorithms include support vector machine (SVM), genetic algorithm (GA), decision tree (DT), random forest (RF), artificial neural network (ANN), and deep learning (DL). One recent study compared the performance of ML models, including Long Short-Term Memory (LSTM) and Support Vector Regression (SVR), with Monod and Haldane models, and the results showed that ML models outperformed the traditional models, especially the light acclimation effect (Yeh et al. 2023). A kinetic-assisted ML was used to predict the growth curve of microalgal biomass, and the proposed ML method had the potential to rapidly estimate algal growth trends and accurately predict biomass with potentially reduced costs (Jiang et al. 2024). Another study adopted a hybrid ML model to predict the impact of light wavelengths on algal growth of five algal genera, including *Chlorella* sp. and achieved high accuracy (R^2^ > 0.96 for total growth and R^2^ > 0.8 for genera-specific and wavelength-based models), suggesting that the integration of empirical data with ML is a robust approach to predict algal growth for industrial applications (Sumanasekara et al. 2025). ML has been shown to accurately describe CO_2_ removal efficiency and *C. vulgaris* growth in a smart photobioreactor, directly contributing to environmental sustainability and the circular economy (Galang et al. 2025). The integration of ML and synthetic biology offered a new solution for biofuel production through improved design of a semi-continuous algal cultivation system to sustain optimal cell growth and minimize mutual shading (Long et al. 2022). These tools are not alternatives to traditional kinetics. Instead, they should be integrated to conventional kinetic equations to resolve complex kinetic parameterization and prediction challenges.

Although many ML models are available, these models face the common challenges of limited available data and scalability issues (Imamoglu 2024). A recent analysis of data mining and ML methods in microalgae systems showed the importance of acquiring data for the development of robust ML models (Freitas et al. 2025). The limitations of ML for optimizing biomass and high-value metabolite production in microalgae were identified as strain screening, growth prediction and optimization, monitoring of growth stages and physiological states, and real-time control (Tang et al. 2026). The authors also claimed that the development of trustworthy ML models was more important than the predictive accuracy of ML models (Tang et al. 2026). Standardization of benchmarks and databases was also considered to be important so ML models can be compared (Tang et al. 2026).

To address the data acquisition and scalability challenges, standardized, open access kinetic database should be developed alongside ML pipelines to esimate kinetic parameters for high-throughput genome-scaling modeling (Toumpe et al. 2025). Such standardization and machine-readable formats are essential for build scalable open access biological knowledgebases (Wood et al. 2026). Furthermore, accurate prediction of algal metabolic flux and growth optimization requires standardization of large physiological databases of various growth conditions to support ML algorithms (Tec-Campos et al. 2026). These open access biological databases should follow the FAIR (Findable, Accessible, Interoperable, Reusable) guidelines and be structured to be compatible for ML applications (Lalonde et al. 2026).

## A proposed decision tree for algal wastewater treatment and resource recovery in the food and beverage industry

**Fig. 6:**
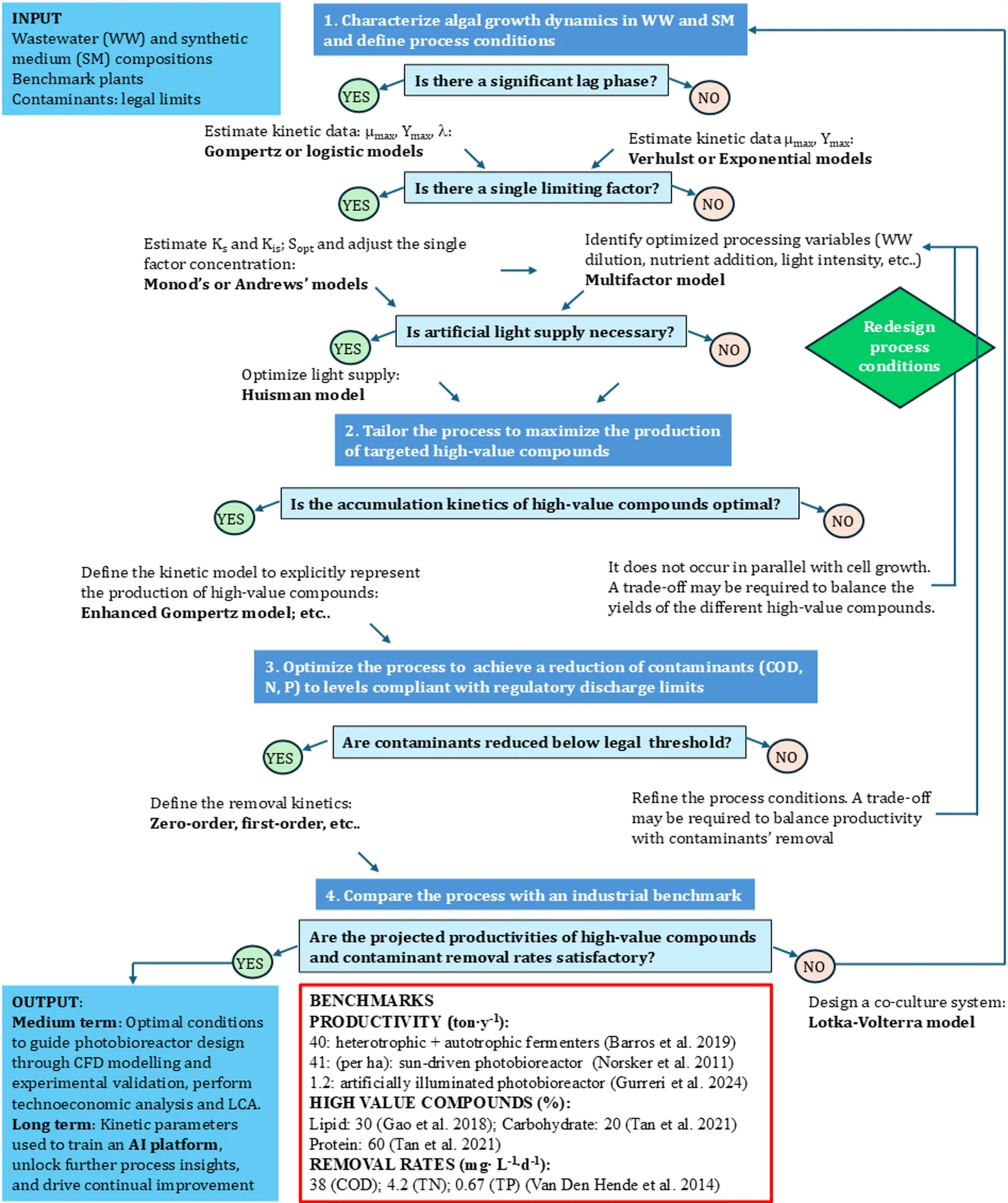
Decision tree based on kinetic models for optimizing algal treatments of food and beverage effluents

A decision tree based on kinetic models for optimizing algal treatments of food and beverage effluents is illustrated in Fig. 6. As a first step, the growth curve in wastewater could be compared with that measured in an optimal synthetic medium. To do so, sigmoidal models, such as the Gompertz and logistic equations, can be used as they offer good and comparable predictive accuracy, although they are relatively complex. They effectively capture the lag phase caused by cell adaptation to the complex C sources present in effluents and provide parameters such as λ and λ’, together with an accurate estimate of μ_max_. The double Gompertz equation can additionally describe sequential nutrient use. When no lag phase is observed, typically because the effluent contains readily accessible C and N sources, or because operational strategies like fed-batch cultivation maintain stable glucose and ammonium levels, simpler models, such as the Verhulst and exponential equations, remain appropriate. Notably, the exponential model is the simplest option, requiring only two consecutive biomass measurements to calculate μ.

If algal growth is driven predominantly by a single limiting factor, Monod and Andrews equations yield physiologically meaningful constants, making them especially useful for optimizing early or late stages of batch cultivation, when the key factor is either abundant or depleted. In contrast, multifactorial models are more suitable for describing the behavior of complex wastewaters with multiple interacting variables, providing valuable support for scaling continuous or fed-batch processes. In systems affected by turbidity or self-shading, the Huisman model becomes particularly relevant, as it accounts for light distribution effects linked to photobioreactor geometry. The Huisman model and the associated concept of critical light intensity have been largely overlooked in small-scale studies, possibly because *Chlorella* competes effectively under light-limited conditions and has a relatively low critical light intensity. Nevertheless, in efforts to increase biomass productivity, the model can predict the maximum steady-state biomass concentration that can be sustained at a given incident light intensity, optical path length, and dilution rate before the transmitted light falls to the critical level.

In the second step, implementing bioeconomy and resource recovery principles requires cultivation strategies designed to maximize the production of value-added compounds. For this purpose, the kinetics of compound accumulation should be analyzed. When this accumulation occurs in parallel with biomass production, the growth curve can be reformulated to explicitly include product formation, as in the enhanced Gompertz equation. Alternatively, if target products’ formation is not aligned with biomass accumulation, multifactor models should be further applied to identify conditions that maximize their productivity. A trade-off becomes necessary whenever multiple products are targeted simultaneously.

In a future industrial context, real-time monitoring of contaminant concentrations will be crucial to regulate influent and effluent flows and ensure consistent compliance with discharge limits. Therefore, as a third step, bioremediation kinetics should be evaluated using Monod, zero-order, or first-order models. If contaminants remain above acceptable thresholds, conditions should again be refined using multifactorial modeling, balancing productivity with contaminant removal efficiency.

Finally, the kinetic data obtained must be projected on an industrial scale and compared to relevant benchmarks. If performance remains unsatisfactory, process redesign should be considered, including the implementation of co-cultures and the characterization of microbial growth dynamics with the Lotka-Volterra model. The use of co-cultures requires evaluating how differences in species-specific light requirements and absorption properties affect the maximum biomass concentration sustainable at the critical light intensity. It is also necessary to determine whether, under conditions designed to maximize biomass productivity, the benefits of metabolic cooperation outweigh the increased competition for light and self-shading. These aspects should first be investigated theoretically by extending the Huisman model to co-cultures and subsequently validated experimentally before scale-up.

In this way, the decision tree, supported by kinetic models initially calibrated at the laboratory scale, can be used to identify the operating conditions—such as C and N source concentrations and, consequently, the degree of effluent dilution, light intensity, and temperature—that favor the achievement of the predefined targets. Once satisfactory performance has been achieved at the laboratory scale, the bioreactor and the associated process-control strategies should be designed with the support of CFD modelling, followed by experimental validation. CFD can be used to design and optimize the bioreactor so that the spatial and temporal distribution of the optimal conditions is as favorable as possible throughout the culture volume, while minimizing gradients, stagnant zones, and unfavorable exposure to light conditions or shear stress. Subsequently, validation experiments can determine whether the identified conditions enable the targets to be achieved at a large scale. The ability to accurately predict the experimental results supports the validity of the kinetic model integrated into the CFD model of the bioreactor. Any discrepancies would instead require the identification of inadequately represented phenomena and, where necessary, the introduction and calibration of correction terms or additional mechanisms in the model equations. For instance, possible mass-transfer limitations can be identified by fitting a modified Gompertz model to growth curves obtained at different mixing rates under otherwise constant conditions. An increase in the apparent maximum growth rate and a shorter lag phase with enhanced mixing would indicate mass-transfer limitations. Although a correction factor can be included in the Gompertz equation, coupling mass-transfer balances with substrate-dependent growth kinetics is preferable for distinguishing transport effects from intrinsic biological limitations. Regarding possible growth limitations due to light attenuation, the Huisman model already accounts for light availability through k_bg_, representing background turbidity caused by non-biomass components, and k, representing biomass-specific light attenuation. Therefore, no additional correction term is required to describe light attenuation within the assumptions of the model.

In parallel, techno-economic analysis and life-cycle assessment should be performed to determine the feasibility of the proposed resource-recovery strategy. In the longer term, the kinetic and experimental data could be used to train an AI-based platform, generate further process insights, and drive continuous improvement.

## Research needs and future direction

The cultivation of *Chlorella* spp. on effluents of the food and beverage industry has been extensively investigated to implement the principles of the circular economy. From the studies summarized above, it was evidenced that for most applications, further research is still needed to increase algal productivity and contaminants’ removal rates. To this aim, integrating kinetic models according to the decision-tree framework will lead to optimization and improvement of the capability to design efficient cultures or co-cultures, comparable to benchmarks.

A deeper insight into the effect of light flow on algal growth in photobioreactors of different geometries and sizes is needed. Hence, future research should also apply the Huisman equation as a fundamental tool to understand how light governs algal growth and shifts their metabolisms. The extension of the Huisman model to synergistic co-cultures and its application to the optimization of large-scale algal cultivation remain largely unexplored and represent an important research gap. In fact, Huisman’s formulations have primarily been applied to competition among photosynthetic populations, whereas synergistic co-cultures involve positive metabolic interactions that may coexist with competition for light and other resources.

Future research should also investigate potentially adverse algal–bacterial interactions. Settled algal cells may undergo bacterial degradation, resulting in biomass losses and nutrient release (Lu et al. 2023). Particular attention should therefore be paid to algae-lysing and algicidal bacteria, which may reduce culture stability and productivity. These interactions should be characterized to develop effective contamination-monitoring and control strategies.

Lastly, considering that nutrient composition affects biomass composition, a better characterization of the algal biomass attainable from specific effluents will lead to the design of targeted applications and hence increase the value of the final product.

## Conclusion

A wide range of strategies has been explored to evaluate the ability of *Chlorella* spp. in both monoculture and co-culture to upgrade effluents from the food and beverage processing sector, with most investigations carried out at laboratory scale. Several of these approaches show promising potential for scale-up to industrial applications. This review is grounded in the idea that modelling is essential for advancing research toward real-world implementation, and its findings contribute to progress in this direction. Benchmark bioprocesses were identified and adopted as reference systems to establish quantitative optimization targets. Building on the diverse approaches critically discussed throughout this review, a decision tree is proposed as an integrated framework for selecting the most appropriate algal treatment and valorization strategy based on effluent characteristics, process objectives, and target value-added products. The kinetic data generated by applying the pathway outlined in the decision tree should support CFD-based bioreactor design and experimental validation, as well as techno-economic analysis and life-cycle assessment. In the longer term, these kinetic and experimental data could be used to train an AI-based platform, generate further process insights, and drive continuous improvement. However, additional efforts are still required to ensure systematic data collection and standardization.

## Supplementary Information

Supplementary material 1.

## Data Availability

The original contributions presented in this study are included in the article. Further inquiries can be directed to the corresponding author.
