# Supplementary material for: Kinetic modelling of Chlorella spp. wastewater treatment and resource recovery in the food industry
Source: Bioresour Bioprocess. 2026 Oct 14;13(1):150. doi: 10.1186/s40643-026-01145-x (PMC13669260; doi:10.1186/s40643-026-01145-x)
Supplement: Supplementary file 1 [file 40643_2026_1145_MOESM1_ESM.docx]

**Figure S1.** Algal growth as modelled by the modified Gompertz equation (left side), the logistic equation (middle) and the Verhulst equation (right side). The equations and their relative physiologically relevant parameters are reported in Table 1.

**Figure S2.** Algal growth as a function of substrate (S) concentration, according to the Andrews model (left side) and as a function of light penetration, according to the Huisman model (right side). The equations and their relative parameters are reported in Table 1.

**Factor X_2_**

**Factor X_1_**

**Output Y_2_**

**Output Y_1_**

**Factor X_2_**

**Factor X_1_**

**Figure S3.** Polynomial quadratic model to optimize the output Y_1_ (left side) or the output Y_2_ (right side) of algal cultivation, by varying the growth factors X_1_ and X_2_, which show positive interaction for the output Y_1_ and negative interaction for the output Y_1_. The equation and the meaning of its parameters are reported in Table 1.

**Figure S4.** Decrease of COD in the effluent according to a zero-order (left side) or a first-order kinetics (right side). The equations and their relative parameters are reported in Table 1.

**i (cell∙mL^-1^)**

**j (cell∙mL^-1^)**

**j (cell∙mL^-1^)**

**i (cell∙mL^-1^)**

**μ_i_**

**μ_i_**

**Figure S5.** Specific growth rate of microorganism “i” cultivated in a consortium with microorganism “j” as modelled by the Lotka-Volterra equation, in the case of positive interaction between “i” and “j” (left side) or negative interactions between “i” and “j” (right side). The Lotka-Volterra equation and the meaning of its parameters are reported in Table 1.
